# Distribution and development of the external sense organ pattern on the appendages of postembryonic and adult stages of the spider *Parasteatoda tepidariorum*

**DOI:** 10.1007/s00427-020-00655-8

**Published:** 2020-02-08

**Authors:** Magdalena Ines Schacht, Martina Francesconi, Angelika Stollewerk

**Affiliations:** 1 School of Biological and Chemical Sciences, Queen Mary University of London, Mile End Road, London, E1 4NS UK; 2 Dipartimento di Biologia, Università di Pisa, Via Volta 4, 56126 Pisa, Italy

**Keywords:** Spiders, External sense organs, Appendages, Morphology, Postembryonic development, Pattern

## Abstract

Spiders are equipped with a large number of innervated cuticular specializations, which respond to various sensory stimuli. The physiological function of mechanosensory organs has been analysed in great detail in some model spider species (e.g. *Cupiennius salei*); however, much less is known about the distribution and function of chemosensory organs. Furthermore, our knowledge on how the sense organ pattern develops on the spider appendages is limited. Here we analyse the development of the pattern and distribution of six different external mechano- and chemosensory organs in all postembryonic stages and in adult male and female spiders of the species *Parasteatoda tepidariorum*. We show that except for small mechanosensory setae, external sense organs appear in fixed positions on the pedipalps and first walking legs, arranged in longitudinal rows along the proximal-distal axis or in invariable positions relative to morphological landmarks (joints, distal tarsal tip). A comparison to other Entelegynae spiders shows that these features are conserved. We hope that this study lays the foundation for future molecular analysis to address the question how this conserved pattern is generated.

**Electronic supplementary material:**

The online version of this article (10.1007/s00427-020-00655-8) contains supplementary material, which is available to authorized users.

## Introduction

Arthropods have a sophisticated sensory system which mediates environmental interactions and supports essential activities such as foraging and mating (Stevens 2013). Besides the well-developed visual sense, arthropods have many small internal and external sense organs, distributed all over the body and all appendages, which process various sensory stimuli (Foelix 2011; Barth 2001). While considerable information is available on the development, distribution and physiology of all types of sense organs in insects, the remaining arthropod groups lack behind.

Here we analyse the distribution and development of the pattern of external sense organs (ESOs) on the appendages of the spider *Parasteatoda tepidariorum*, with specific focus on the chemosensory (CS) setae, which are understudied in spiders. Previous publications suggest that CS setae are more abundant on the anterior appendages (Foelix 1970; Pfreundt and Peters 1981; Ganske and Uhl 2018) and we therefore investigate here the external sensory equipment of the pedipalps and 1st walking legs (L1). Spiders have six pairs of appendages. The most anterior pair are the short chelicerae, which inject the venom. The next pair, the pedipalps, are used for food uptake but in male spiders also for transferring sperm into the genital opening of females. The anterior pairs are followed by four pairs of walking legs, which are made of seven podomeres: coxa, trochanter, femur, patella, tibia, metatarsus and tarsus (Foelix 2011). The pedipalps are morphologically similar to the walking legs, but lack the metatarsal podomere, and exhibit an additional basal outgrowth (gnathendite) that functions as a lateral wall of the preoral cavity (Foelix 2011).

In arthropods, ESOs originate from precursor cells in the epidermis, which form cuticular specializations such as bristles, sockets and slits, in addition to the neural part of the sense organ (Hartenstein 2005). The sensory neurons transmit the sensory stimuli to the central nervous system where the information is processed and transformed into a neural output such as a behavioural response (Hallberg and Hansson 1999; Hartenstein 2005; Chapman et al. 2013). Similar to the other arthropod groups, spiders exhibit a large variety of sensilla shapes. Here we focus on the six best described sensilla: the mechanosensory (MS) setae, trichobothria, slit sensilla, lyriform organs, tarsal organs and CS setae (Table 1).

**Table 1:**
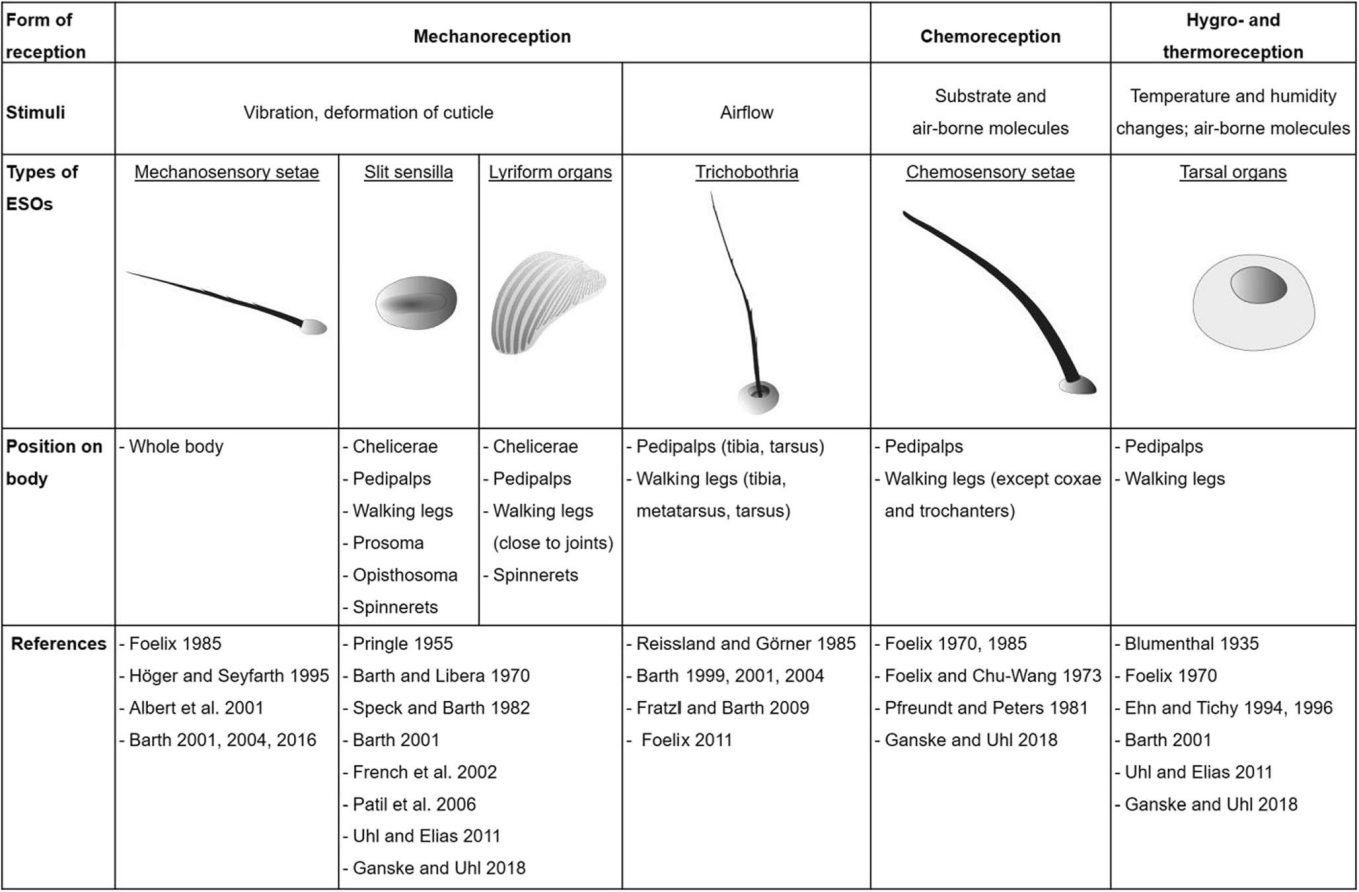
Morphology and function of external sense organs in spiders. The table summarizes the published information on the structure and function of the six sense organs analysed here

MS setae are touch sensitive, have a tapered tip and can be short, long and slender (hair-like), or sturdy (bristle-like). Many exhibit a serrated surface (Table 1) but smooth MS setae have been described as well (Eckweiler et al. 1989). Slit sensilla can only be found in arachnids and appear in three categories: (1) isolated single slits, (2) groups of scattered slits and (3) lyriform organs composed of many parallel individual slits (Barth 2001). Slit sensilla respond to mechanical strain of the cuticle, which can be triggered by muscular activity, haemolymph pressure, gravity and substrate vibrations (Barth 2001). Trichobothria are the air movement sensors of spiders and unique to arachnids. They have a distinct cup-like socket and a long hair-like cuticular outgrowth (Table 1; Barth 2001).

The tarsal organ is an open pit located on the dorsal tarsus. It contains several sensilla with single pores at the tip suggesting an olfactory function but electrophysiological recordings also indicate responses to humidity and thermal stimuli (Barth 2001). The CS setae (also called tip-pore sensilla) of spiders exhibit a blunt end with an open pore, are striated and slightly curved and arise at a steep angle from the socket (Pfreundt and Peters 1981). They are the only type of CS setae described so far in spiders and it is therefore assumed that they are used both for gustatory and olfactory reception (Barth 2001; Ganske and Uhl 2018).

Here, we describe the distribution of these 6 types of sensilla in the pedipalps and L1 of *P. tepidariorum* in all postembryonic and adult stages and both sexes. The aim of the paper is to contribute to our understanding of the evolution of sense organ patterns and to provide the morphological basis for future molecular genetic studies in this model spider species.

## Results and discussion

### Six external sensory organs can be identified in *P. tepidariorum* appendages

**Fig. 1:**
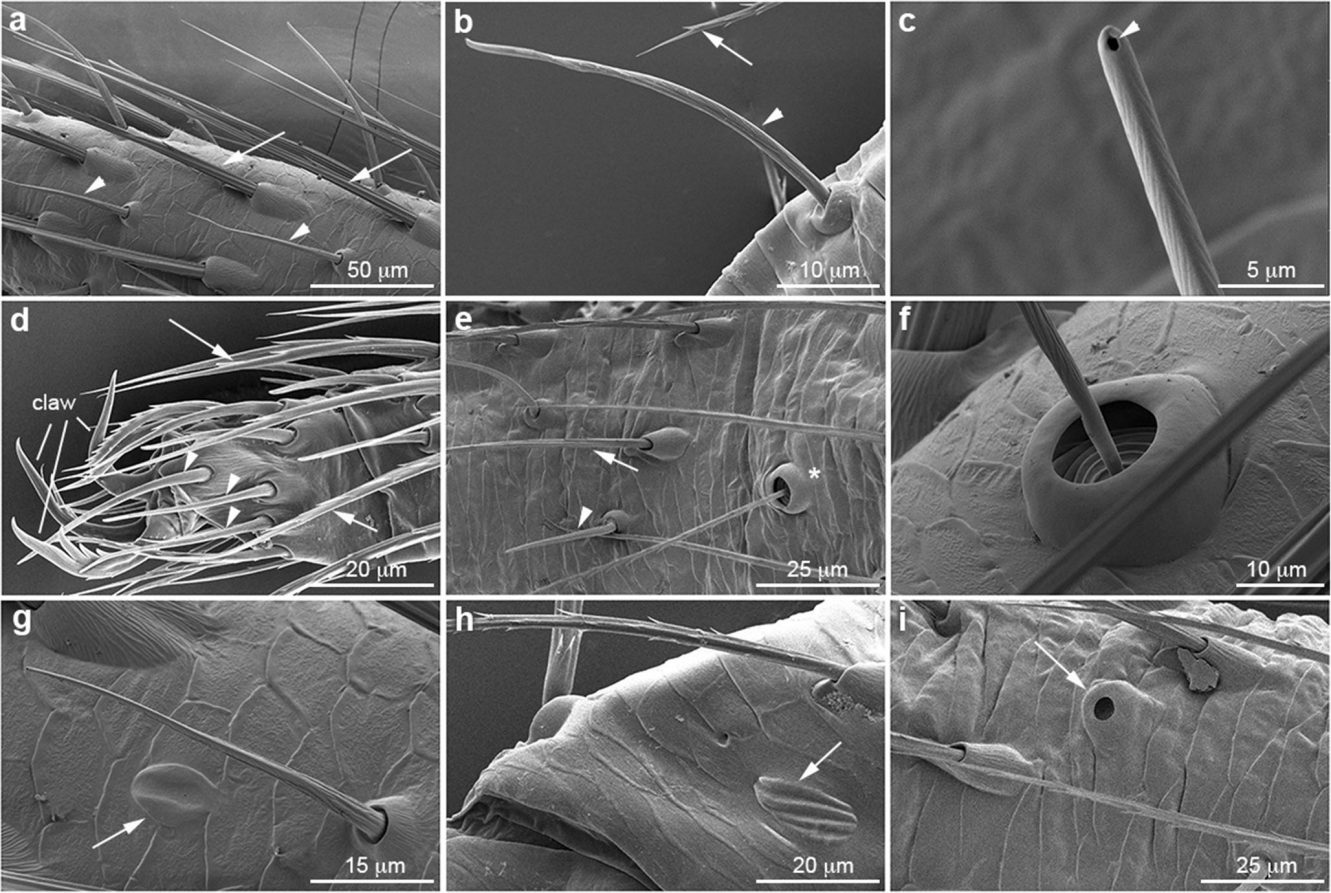
Morphology of external sensory organs in *P. tepidariorum*. Scanning electron micrographs: distal is towards the left. **a**, **c**, **f**–**i** Adult L1. **b**, **d**, **e** 1st instar L1. **a** Mechanosensory (MS) setae are inserted at flat angles (arrows), while chemosensory (CS) setae are positioned at steep angles (arrowheads). MS and CS sockets can be distinguished by the different positions of their mounting holes, which open towards distal in MS setae and vertically in CS setae. MS setae show a longitudinal grooved profile (arrows), while CS setae have shallow spiral indentations (arrowheads). **b**, **c** High magnification of CS setae showing the shallow spiral indentations and the open pore at the tip (arrowheads). MS setae are equipped with spines (arrow in **b**). **d***P. tepidariorum* has a claw at the tip of the tarsal podomere. The arrow and arrowheads point to MS and CS setae, respectively. **e** All external sense organs described here are already visible in the 1st instar (arrow, MS seta; arrowhead, CS seta; asterisk: trichobothrium. **f** High magnification of the typical cup-shaped socket of a trichobothrium. **g** Structure of a single slit sensillum (arrow). **h** Lyriform organ with three slits (arrow). **i** The arrow points to the single tarsal organ, which is located on the dorsal-posterior side of each tarsal segment

All six types of sensilla are present on the appendages of the 1st instar and throughout postembryonic development (Suppl. Fig. 1). MS and CS setae can be distinguished by their different surface structures. MS setae show a longitudinal grooved profile with spines, while CS setae appear striated due to shallow spiral indentations and have a pore at the tip (Fig. 1a–c). Furthermore, the different insertion angles of MS and CS setae (shallow and steep, respectively) are reflected in the different positions of the mounting holes in the sockets. In MS setae, the sockets open towards distal, while they open vertically in CS setae (Fig. 1a, b). The first CS setae appear at the tip of the tarsus, close to the claw and can clearly be distinguished from the MS setae by the above described morphological features (Fig. 1d). Trichobothria are only present on the tibia and metatarsus and exhibit the typical cup-like socket (Fig. 1e). Similar to MS setae, the cuticular outgrowth shows a grooved surface with spines but it arises at a steep angle and is only half as wide as the MS setae. Single slit sensilla are scattered over all podomeres and vary in size (8–20 μm) (Fig. 1g). The lyriform organs, which are composed of several slit sensilla, are always located close to the joints (Fig. 1h). The tarsal organ of *P. tepidariorum* shows a dome-like shape and a central pit (Fig. 1i).

The sense organs seem to develop in fixed positions after each moult. This is most evident in early postembryonic stages and in the pattern of sense organs that appear in low numbers such as trichobothria, lyriform organs and the tarsal organ (Suppl. Fig. 1). MS and CS setae also appear in the same segments and areas in the different stages analysed; however, morphological changes due to appendage outgrowth and their increasing number make it impossible to identify most of the individual sense organs in different stages. In the following, we will present and discuss our results by sense organ type.

### The distribution and number of mechanosensory setae varies between individuals and stages

**Fig. 2:**
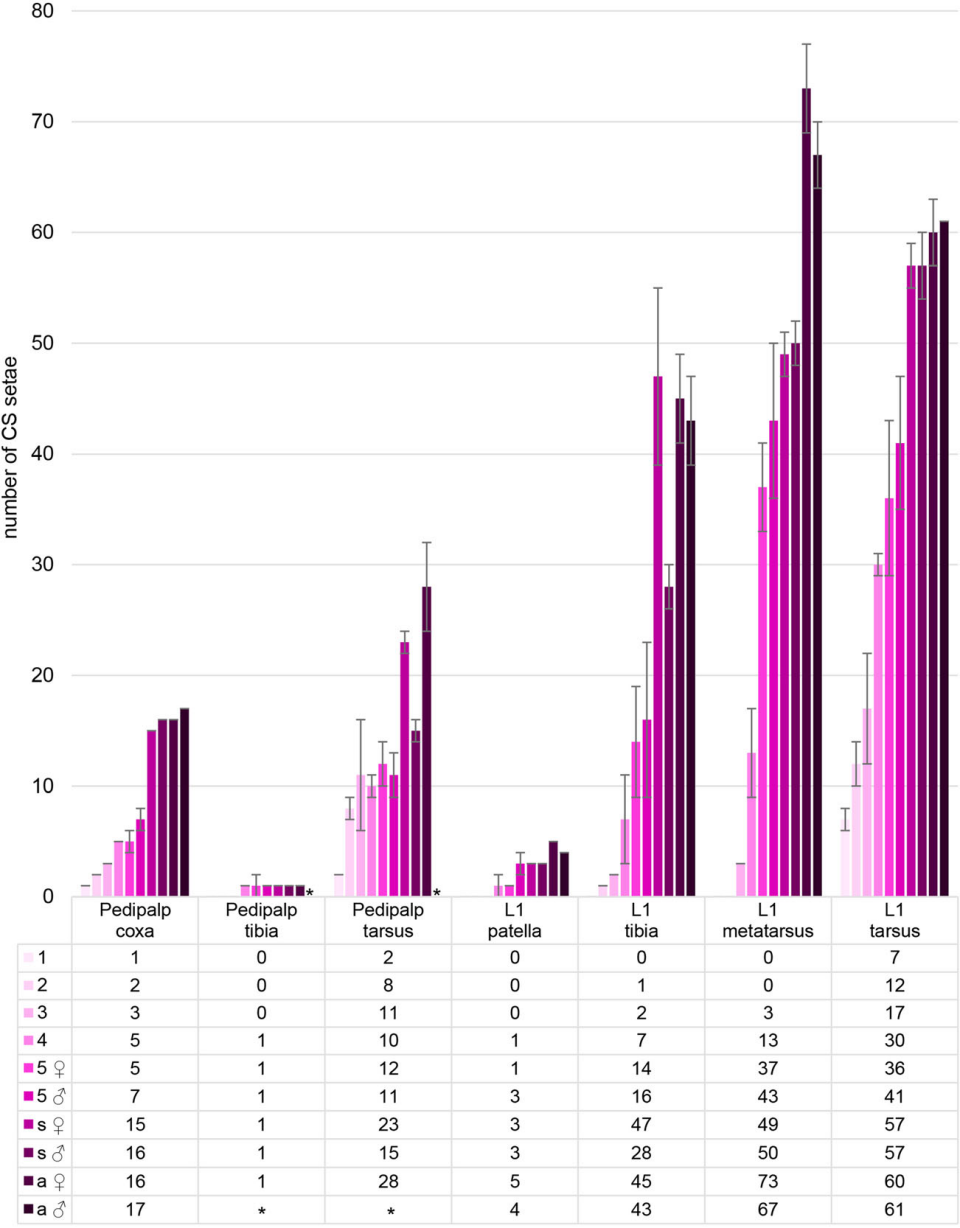
Quantification and distribution of chemosensory organs. The figure shows a comparison of the CS setae on the podomeres of pedipalps and L1 in all postembryonic stages and in female and male adults. We did not count the setae on the tibia and tarsus of the adult male pedipalp (asterisks) because these podomeres form a darkly pigmented copulatory organ which makes the visibility of setae difficult in the light microscope (see main text for details). 1 to 5, 1st to 5th instars; s, subadult; a, adult

**Fig. 3:**
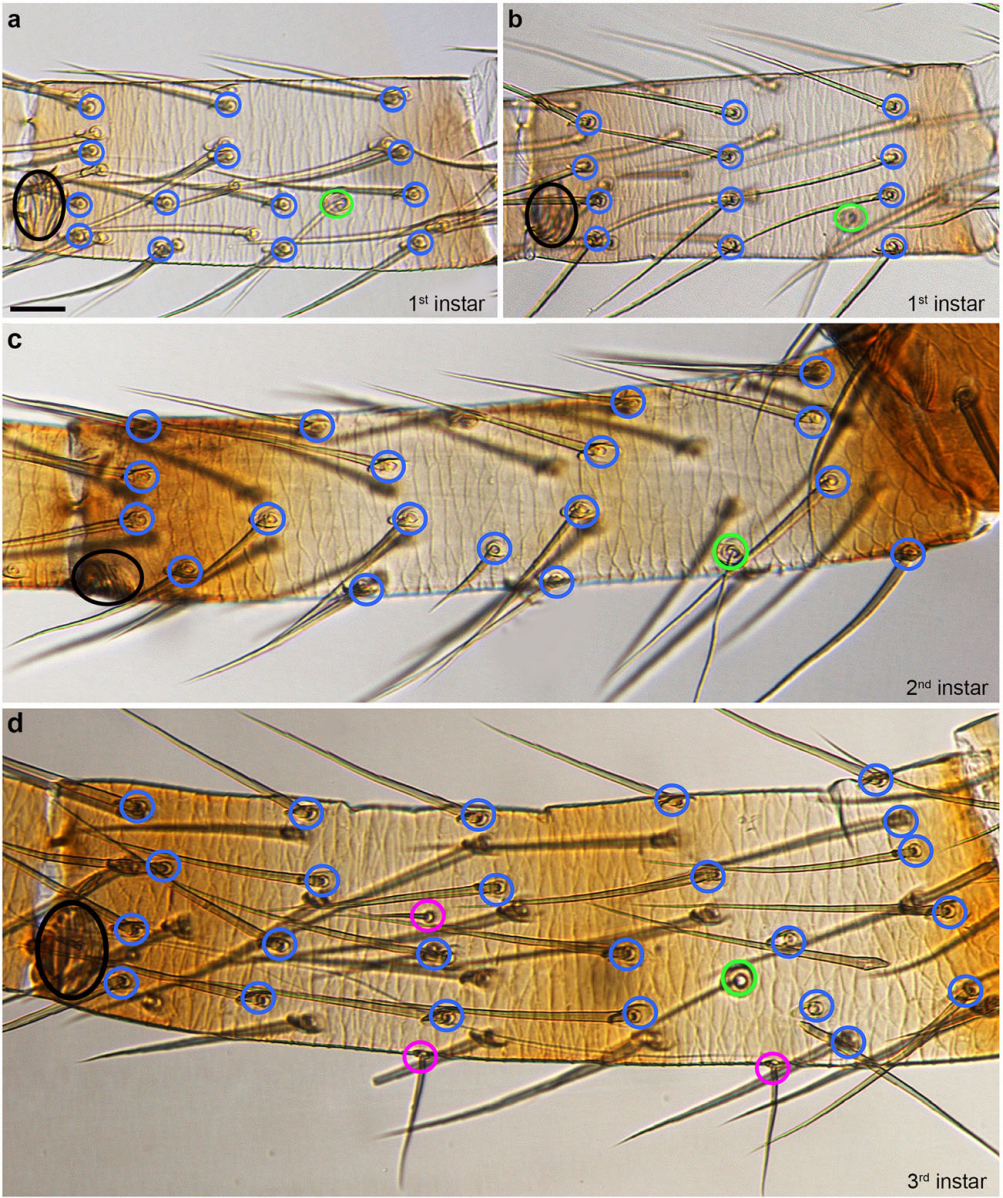
Distribution of mechanosensory sensilla in the metatarsus. Light micrographs, dorsal view: distal is towards the left, anterior towards the top. The blue rings indicate the sockets of MS setae, the green rings the trichobothria, the magenta rings the CS setae and the black rings the lyriform organs. Please note that the setae from the ventral side are visible due to the transparent nature of the cuticle. **a**, **b** The MS setae of 1st instars show similar but not identical ring-like arrangements in the metatarsi from different individuals, while the lyriform organs and trichobothria are located in the same relative positions. **c**, **d** The distribution of MS setae shows significant differences between stages, which is also linked to considerable longitudinal growth. Scale bar in **a**: 15 μm in **a**–**d**

MS setae are the most abundant type of ESOs (Fig. 2; Suppl. Fig. 2a,b; Suppl. Table 1). They appear to be arranged in rings around the podomeres from the 1st instar onward but they are not always exactly aligned (Fig. 3). There are also significant differences in the number and arrangement of MS setae between individuals and instars (Fig. 3a, b; Suppl. Fig. 2a,b). The differences increase with progressing development, in particular in podomeres with large numbers of MS setae (femur, tibia, metatarsus) (Figs. 2 and 3; Suppl. Table 1). It is therefore (mainly) not possible to individually identify MS setae in the pedipalp and L1 of *P. tepidariorum*. A comparison between instars is additionally complicated by the considerable longitudinal growth of the femur, tibia, metatarsus and tarsus during development (compare Fig. 3a, c, d for metatarsus). Furthermore, there are considerable differences in the number of MS setae between adult male and female *P. tepidariorum*. For example, the female adult has 1612 MS setae on L1, while the male exhibits only 981 MS setae on the same leg (Suppl. Fig. 2b). The sex differences are already visible in the subadult stages and also apply to the pedipalps (Suppl. Fig. 2a). They are most likely due to the bigger size of the females (McGregor et al. 2008). However, we identified three exceptionally large MS setae, one on the patella and two on the tibia, which we used as landmarks and which can be followed through all postembryonic stages into the adult (Suppl. Fig. 1). Our results are in line with a study on MS setae (tactile hairs) in *Cupiennius salei* (Höger and Seyfarth 1995), which describes uniquely identifiable large MS setae (long tactile hairs) on the ventral side of proximal podomeres (distal podomeres were not analysed) in postembryonic to adult stages. Here they seem to be involved in the characteristic body raising behaviour, which spiders exhibit to navigate obstacles (Höger and Seyfarth 1995). Similar to our results, the remaining (smaller) MS setae cannot be individually identified throughout postembryonic stages.

### Chemosensory sensilla are arranged in longitudinal rows on the 1st walking leg

**Fig. 4:**
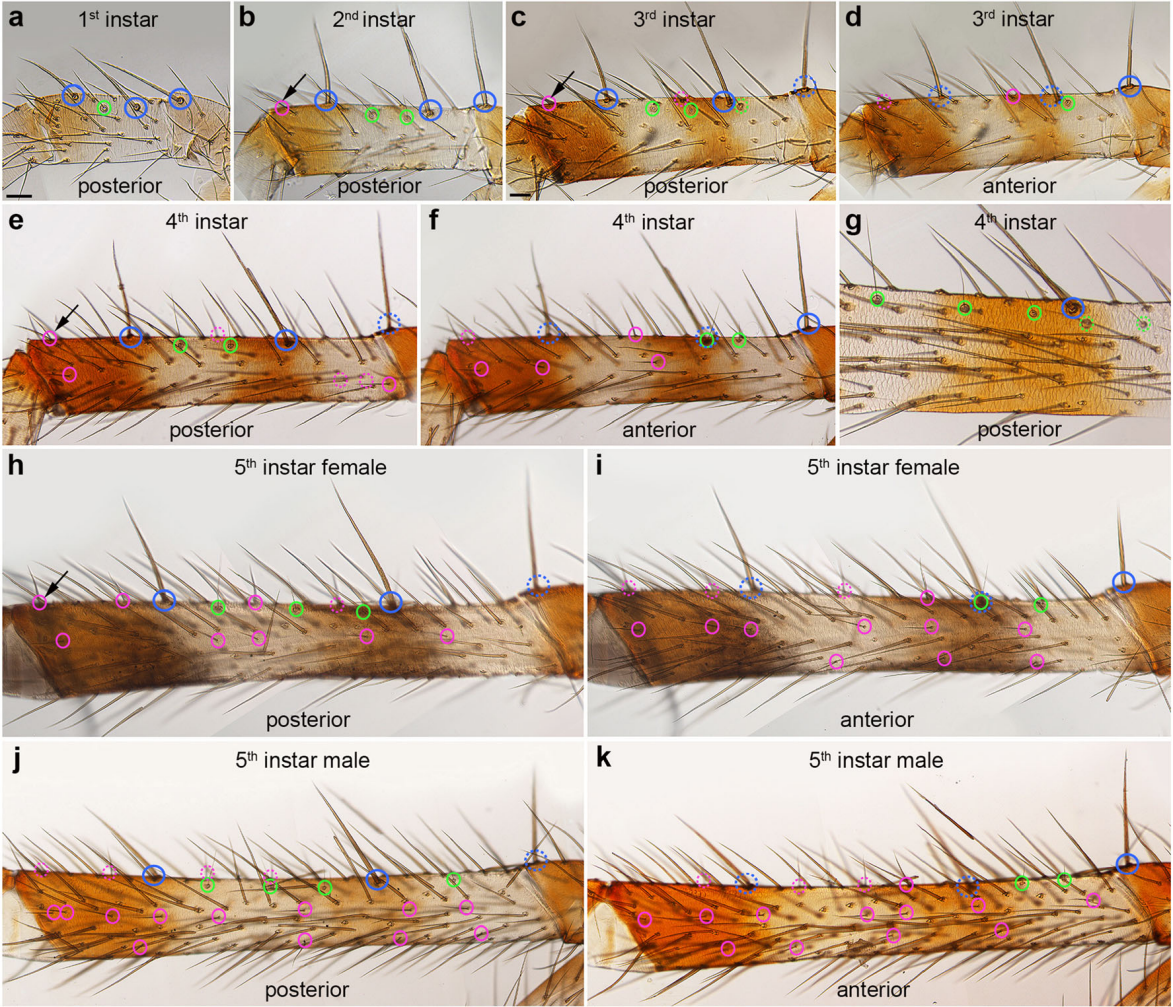
Comparison of the positions of trichobothria and chemosensory sensilla on the tibia of the 1st walking leg in the 5 subsequent postembryonic stages. Light micrographs: anterior and posterior views as indicated; dorsal is towards the top. The blue rings indicate the position of large landmark MS setae which are located at unique positions throughout postembryonic development and in adults. The green rings indicate trichobothria and the magenta rings CS setae. The dashed lines indicate sensilla from the opposite side that are visible due to the transparency of the cuticle; arrows in **b**, **c**, **e**, and **h** indicated CS setae that are identifiable through several stages. **a** The first tibial trichobothrium appears at the 1st larval instar between the large proximal and distal tibial MS on the posterior-dorsal side of L1. **b** In the 2nd instar, a second trichobothrium is visible next to the previous one and the first CS setae appears close to the tibia-metatarsal joint. **c**, **d** In the 3rd instar, an additional trichobothrium is visible on the anterior-dorsal side and a second CS seta is visible between the large tibial MS setae. **e**–**g** Two to three trichobothria are present on the posterior side and two on the anterior side. CS setae start to form the median CS row. **h**–**k** Three to four trichobothria are visible on the posterior side of 5th instar males and females. The CS setae have formed a ventral row. dCS, dorsal row of CS setae; amCS, anterior-median row of CS setae; pmCS, posterior-median row of setae; vCS, ventral row of CS setae; d, dorsal; dl, distal; prox, proximal; v, ventral. Scale bars: **a**, 20 μm in **a**, **b**, **e**–**k**; **c**, 20 μm in **c**, **d**

**Fig. 5:**
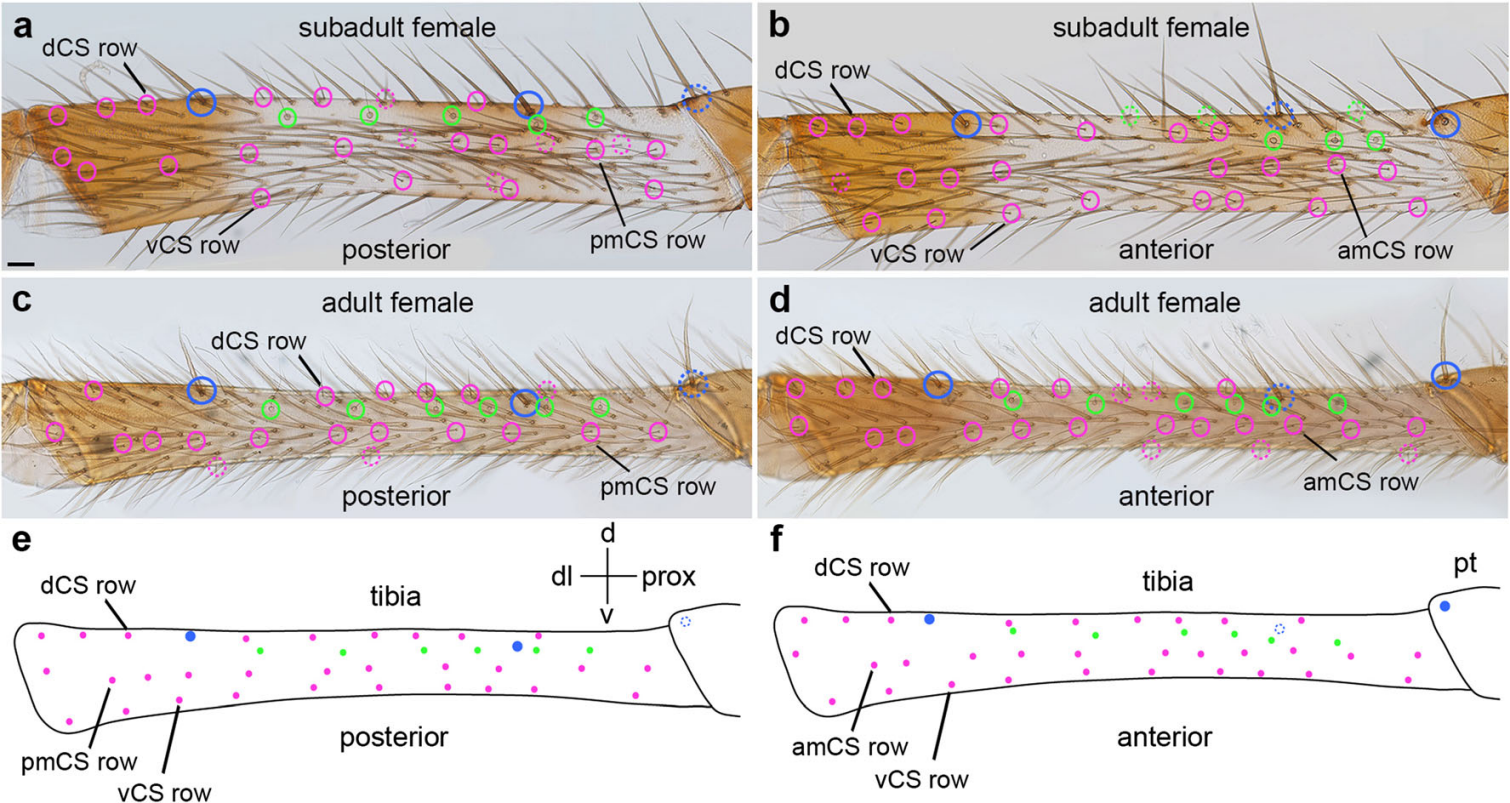
Distribution of trichobothria and chemosensory sensilla in the tibia of subadult and adult female 1st walking legs. Light micrographs (**a**–**d**) and schematic drawings of the tibia. Orientation of the preparations, colouring of the rings and abbreviations are the same as in Fig. 3. **a**, **b** There are 8 trichobothria, 5 on the posterior side and 3 on the anterior side. Three rows of CS setae are visible on either side of the subadult tibia, dCS, amCS or pmCS and vCS (dorsal, anterior-medial or posterior-medial and ventral, respectively). **c**, **d** In adult females, the number of trichobothria has increased to 6 on both sides and the anterior and posterior dorsal and ventral CS rows have intercalated to form single CS rows. **e**, **f** Distribution of the trichobothria and CS setae based on a single female. Scale bar in **a**: 50 μm in **a**, **b** and 75 μm in **c**, **d**

**Fig. 6:**
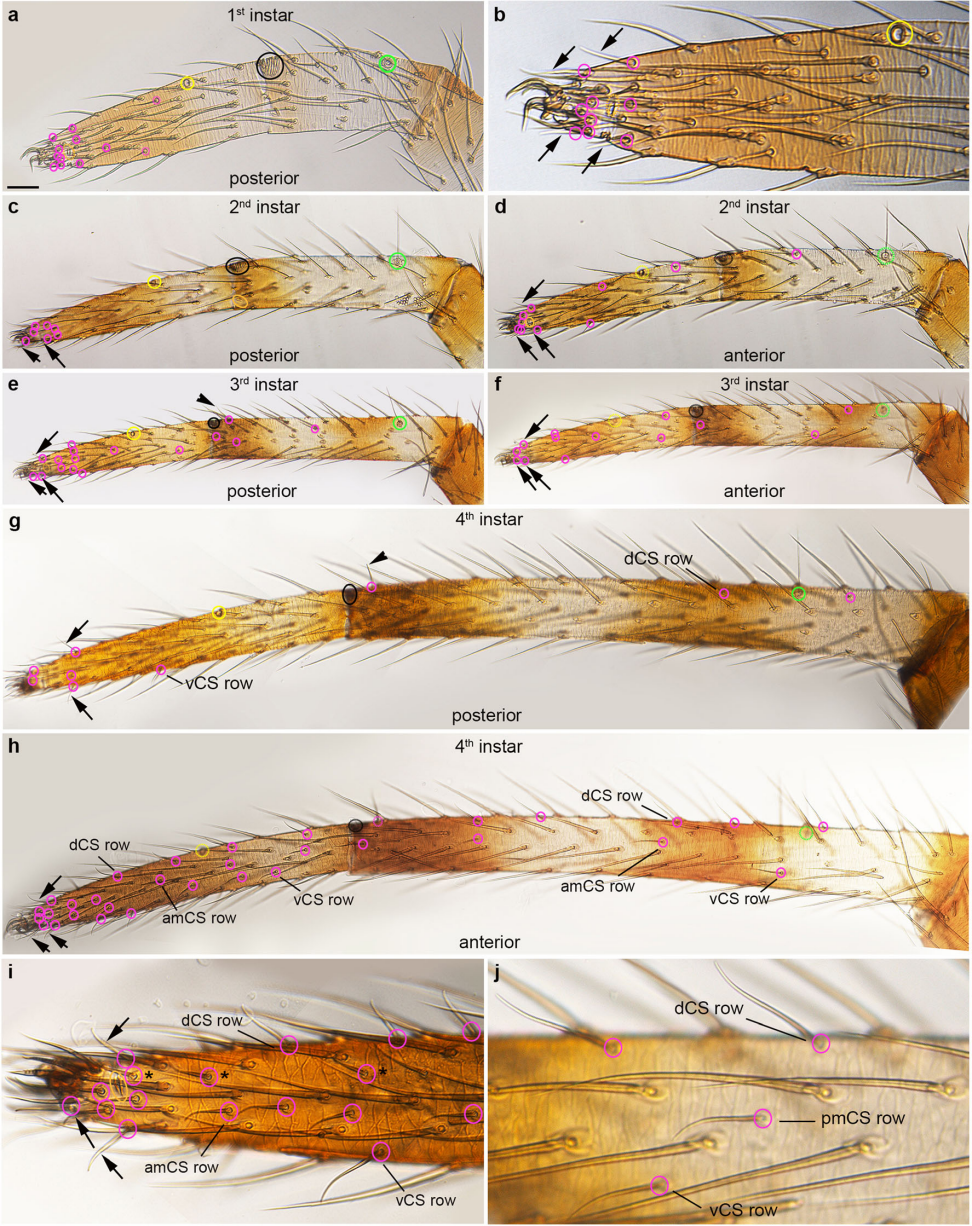
Distribution of trichobothria and chemosensory sensilla in the metatarsus and tarsus of the 1st walking legs of 1st to 4th instars. Light micrographs; orientation of the preparations, colouring of the rings and abbreviations are the same as in Fig. 3. **a**, **b** The first set of tarsal CS setae is already visible in the 1st instar. The positions of some of the CS setae (tarsus: arrows in **b**–**i**; metatarsus: arrowheads in **e**, **g**) are similar in different preparations and can be followed through developmental stages. The tarsal organ (yellow ring) and metatarsal posterior-dorsal lyriform organ (black ring) and single trichobothrium (green ring) are fixed landmarks throughout the postembryonic stages. **c**, **d** The first metatarsal CS seta appears in the 2nd instar (magenta ring) in a dorsal-anterior position in the middle of the segment. **e**, **f** Additional CS setae are visible in the 3rd instar and start to establish a dorsal, medial and ventral row, similar to the arrangement on the tibia. **g**, **h** Many additional CS setae have formed in the proximal part of the tarsus, arranged in a dorsal, medial and ventral row. **i** In the distal part of the tarsus, CS setae (asterisks) form an additional medial row (anterior view). **j** High magnification of the medial-posterior part of the metatarsus showing the arrangement of CS setae in a dorsal, medial and ventral row. Scale bar in **a**: 20 μm in **a**; 12 μm in **b**; 30 μm in **c**, **d**; 70 μm in **e**, **f**; 40 μm in **g**, **h**; 15 μm in **i**, **j**

**Fig. 7:**
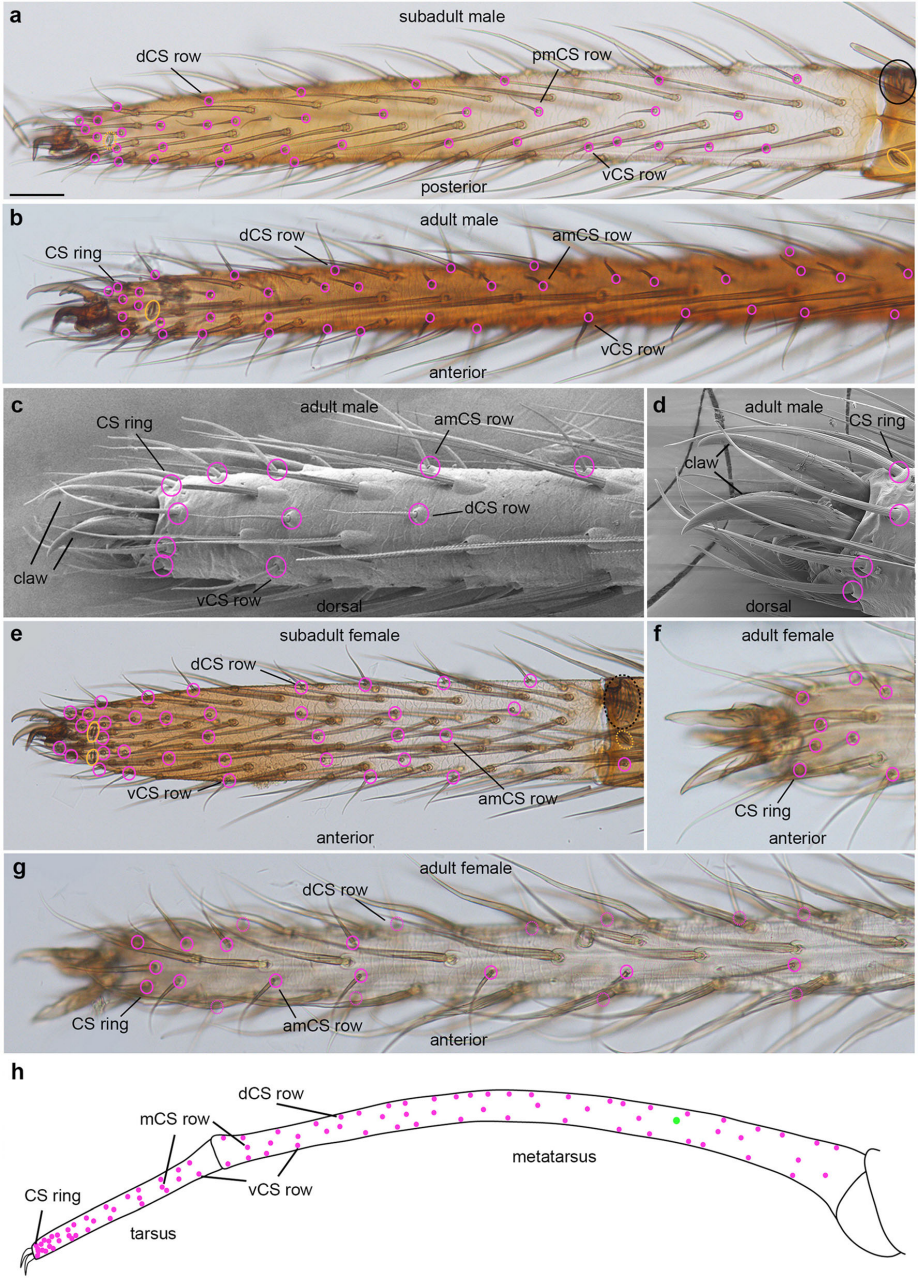
Distribution of trichobothria and chemosensory sensilla in the metatarsus and tarsus of the 1st walking legs of subadults and adults. Light micrographs (**a**, **b**, **e**–**g**), scanning electron micrographs (**c**, **d**) and schematic drawing (**h**); orientation of the preparations, colouring of the rings and abbreviations are the same as in Fig. 3 and as indicated in the panels. **a** In the proximal part of the subadult L1, the CS setae are arranged in 3 rows. In the distal part, additional medial CS setae are visible. **b** In the adult, the CS setae intercalate to form a single dorsal and ventral row, in addition to the anterior and posterior median rows. **c**, **d** The CS setae are arranged in a ring-like shape around the distal tip of the leg, i.e. the base of the claw. **e** In the female subadult preparation, the pattern of CS setae appears denser compared to the subadult male; however, this correlates with the shorter length of the tarsus and the average number of CS setae is the same. **f**, **g** In the extended adult female tarsus, the arrangement of the CS setae is the same as in the male. **h** Similar to the tibia, 3 rows of CS setae are visible in the tarsus and metatarsus, dCS, mCS, vCS (dorsal, medial and ventral, respectively). In the distal part of the tarsus, a distal CS ring and additional medial CS setae are visible. Scale bar in **a**, 40 μm in **a**; 70 μm in **b**; 30 in **c**; 20 μm in **d**; 60 μm in **e**; 40 μm in **f**; 70 μm in **g**

Chemosensory (CS) setae are present on the distal most 2–4 podomeres of the pedipalp and L1, respectively, in addition to the pedipalp coxa (Fig. 2; Suppl. Table 1). The first CS setae are visible on the tip of the tarsus in the 1st instar, both in the pedipalp and L1. After each moult, they gradually increase in number and spread to more proximal segments (Fig. 2; Suppl. Table 1). There is a dorsal row of only 5 CS setae on the L1 patella of adults, both in males and females (data not shown), which we did not further analyse here, while the remaining CS setae can be found on the three distal most podomeres. We present the analysis of CS setae arrangement by podomere, from tibia to tarsus of L1 (Figs. 4, 5, 6, and 7; Suppl. Fig. 3), followed by the analysis of the pedipalpal podomeres.

On the tibia of L1, the first couple of CS setae appear in fixed positions on the dorso-posterior side close to the metatarsal joint (2nd instar) and on the dorso-anterior side in the middle of the podomere between the landmark MS setae (3rd instar) (Fig. 4a–d). In the following stages, additional CS setae appear in dorsal, medial and ventral positions on both sides (anterior and posterior) of the leg, establishing longitudinal rows along the proximo-distal axis (Fig. 4h–k). Although the distribution is similar between individuals, stages and sexes, only few CS setae are individually identifiable due to their proximity to landmarks (i.e. joints, large MS setae; e.g. arrows in Fig. 4b, c, e, h). Both in males and females, the three longitudinal CS setae rows on each side of the leg are visible until the subadult stage (Fig. 5a, b; Suppl. Fig. 3a, b); however, in the adult, the CS setae of the anterior dorsal and posterior dorsal rows intercalate to form a single longitudinal row (Fig. 5c–f; Suppl. Fig. 3c-g). The same is the case for the ventral anterior and posterior rows. This might be due to the considerable elongation and simultaneous narrowing of the podomeres in the adult moult.

In the 1st instar, most of the tarsal CS setae are located at the distal tip (Fig. 6a, b). The first metatarsal CS seta appears on the anterior-dorsal side in the middle of the podomere in the 2nd instar (Fig. 6c, d). Similar to the tibia, some of the CS setae can be identified in different stages and preparations (Fig. 6b–i). In the 3rd and 4th instars, additional CS setae establish dorsal, medial and ventral rows on both sides of the leg, similar to the arrangement on the tibia (Fig. 6e–j). At the distal tip of the tarsus, CS setae form an additional row on either side of the leg, resulting in a higher density of CS setae compared to all other areas of the leg (Fig. 6i). The number of CS setae further increases into the subadult stage (Fig. 2; Suppl. Table 1) but the pattern remains the same (Fig. 7a, e). Similar to the tibia, the dorsal and ventral tarsal and metatarsal CS rows intercalate to form single longitudinal rows (Fig. 7b, c, g, h). In addition, a ring of 8 CS setae surrounds the distal tip of the adult tarsus, both in males and females (Fig. 7b–d, f–h).

The number and distribution of L1 CS setae are comparable to the pattern in other spider species. Similar to *P. tepidariorum*, the CS setae of the walking leg are mainly found on the distal podomeres (tibia, metatarsus and tarsus) in all Entelegynae spiders analysed (e.g. *Pardosa prativaga*; e.g. Pfreundt and Peters 1981; Foelix 1970; Ganske and Uhl 2018). Furthermore, in all species, the highest density of CS setae is visible on the tarsus, followed by the metatarsus. The CS setae are arranged in longitudinal rows on all three distal podomeres in all species analysed, although variations in the number of rows (two to eight) have been described between podomeres and between species. While *P. tepidariorum* has four rows on all three distal podomeres, *P. prativaga*, for example, which exhibits one of the largest numbers of CS setae (300 on L1), has three CS rows on each side of the leg in addition to a dorsal and a ventral row (Pfreundt and Peters 1981). In contrast, in *Coelotes terrestris*, all dorsal and lateral rows are missing in the tibia and in *Philodromus aureolus*, there are differences in the number of rows between tarsus/metatarsus and tibia (Pfreundt and Peters 1981).

### Tarsus and coxa show the highest density of chemosensory setae in the pedipalp

**Fig. 8:**
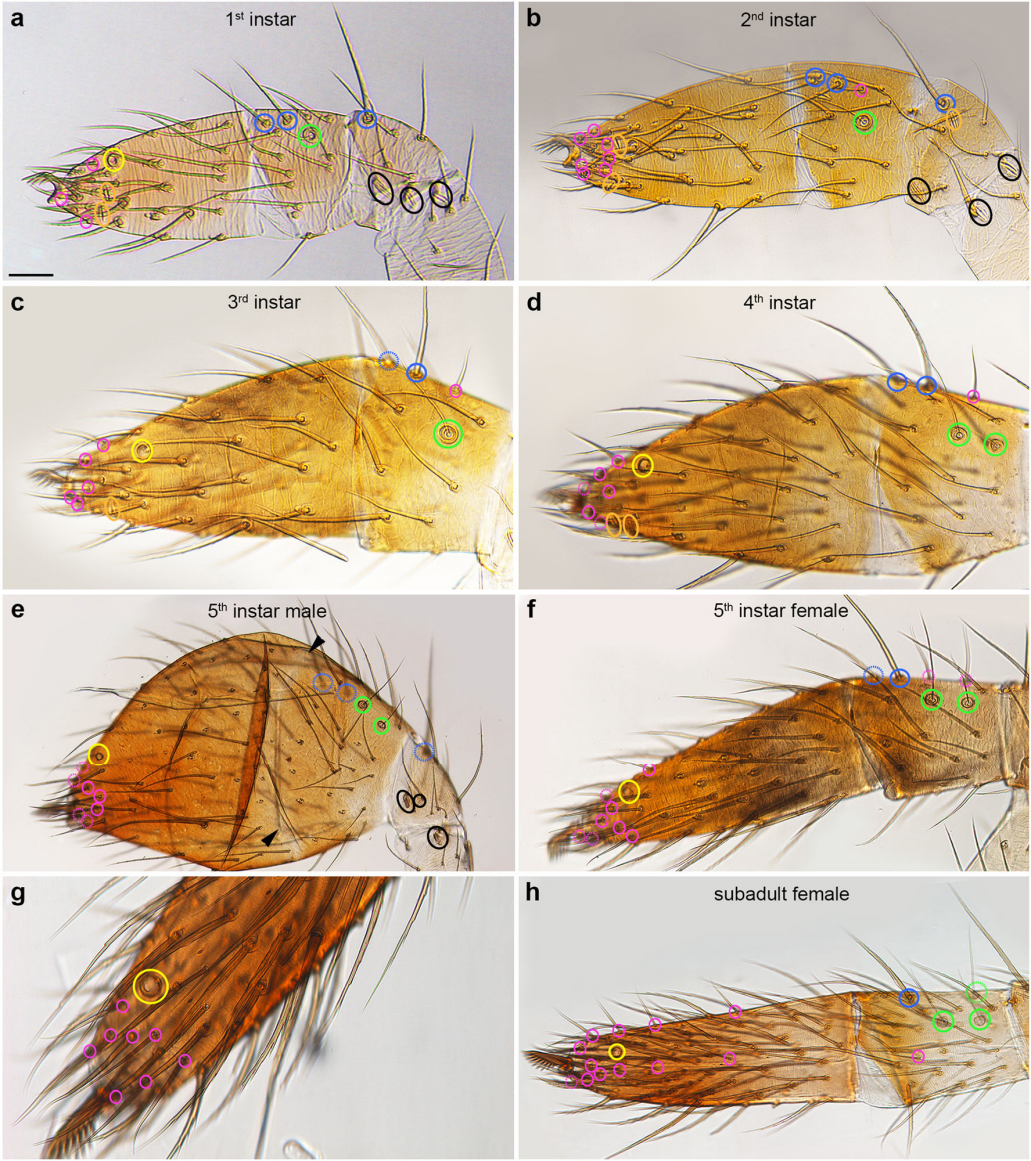
Distribution of trichobothria and chemosensory sensilla in the tibia and tarsus of pedipalps of 1st to 5th instars and subadult females. Light micrographs, posterior views; colouring of the rings and abbreviations are the same as in Fig. 3. **a** In the 1st instar, 1–3 CS sensilla are visible at the tip of the tarsus. A single trichobothrium is present on the tibia. Similar to L1, large landmark MS sensilla are visible, which are located at unique positions throughout postembryonic development and in adults. **b** Additional CS setae surround the tip of the tarsus. A single CS seta is present on the tibia. This sensillum can be followed through the remaining postembryonic stages due to its position next to the landmark MS setae and trichobothria. **c**, **d** The number of CS setae does not change significantly during the 3rd and 4th instars. An additional trichobothrium is visible on the tibia in the 4th instar. **e**, **f** In the 5th instar, male and female pedipalps can clearly be distinguished by the different shapes of the distal segments. The arrowheads in **e** indicate the border between the tarsal and tibial podomeres. **g** High magnification of the distal tip of the tarsus of a subadult female. **h** An additional trichobothrium (dashed green ring) is present on the tibia. Scale bars in **a**: 20 μm in **a**; 30 μm in **b**; 25 μm in **c**; 35 μm in **d**; 60 μm in **e**, **f**; 40 μm in **g**; 85 μm in **h**

**Fig. 9:**
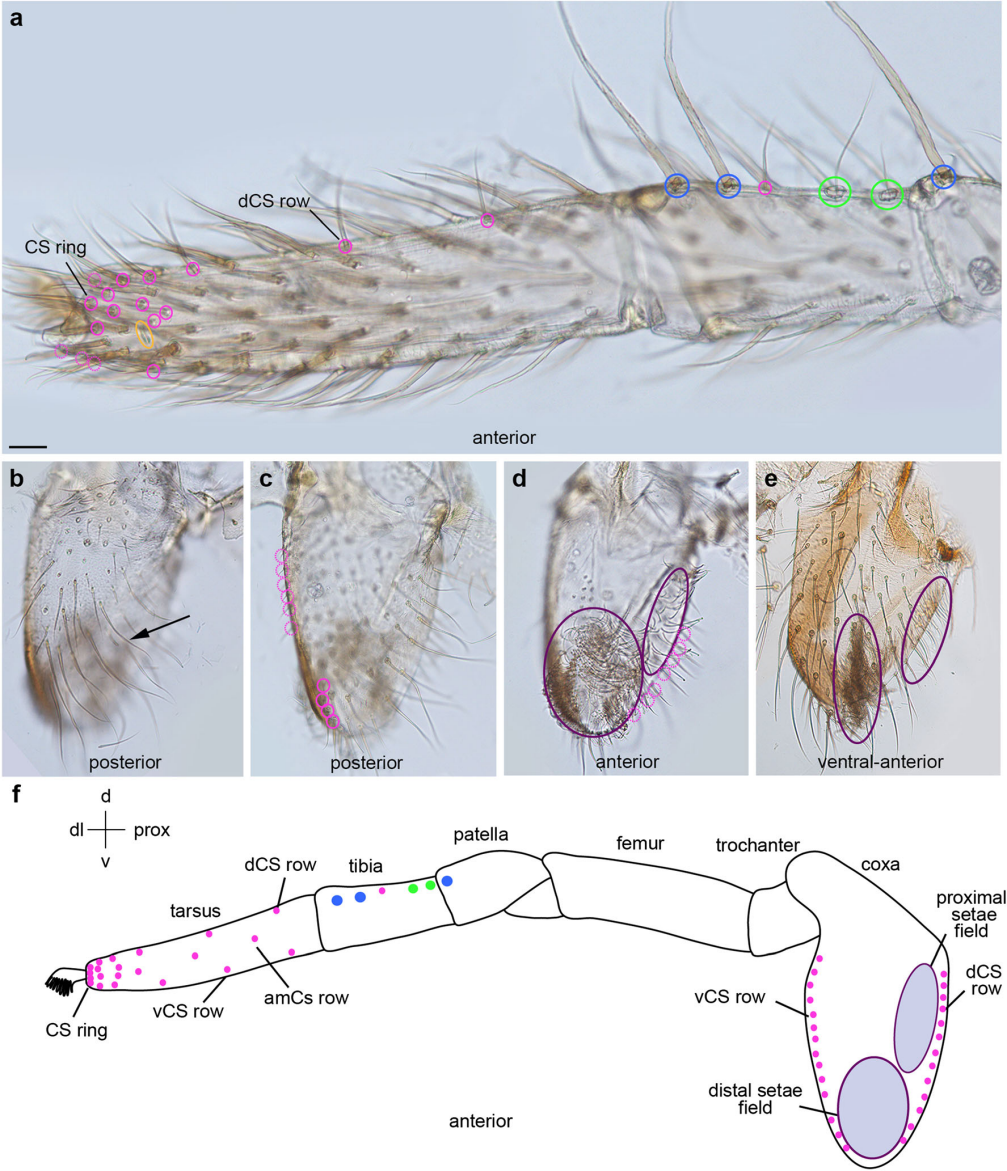
Distribution of trichobothria and chemosensory sensilla in the tarsus, tibia and coxa of adult female pedipalps. Light micrographs (**a**–**e**) and schematic drawing (**f**); orientation of preparations as indicated; colouring of the rings and abbreviations are the same as in Fig. 3; purple rings indicate fields of setae with unknown identity. **a** CS setae are widely spaced in the proximal part of the tarsus. The arrangement of the CS setae in the distal part of the tarsus is similar to L1. On the tibia, the two anterior-dorsal trichobothria, the single CS seta and the landmark MS setae are arranged in a longitudinal row. **b**–**d** Posterior to anterior views of the same coxa. The arrow in **b** indicates long slender setae with tapered tips. The ventral row of CS setae becomes visible in deeper posterior layers. **d** A distal and a proximal setae field are visible on the anterior side of the coxa. The setae are long and curved. A longitudinal row of CS setae is present on the dorsal side of the coxa. **e** The two setae fields are visible in the subadult female but are expanded in the adult (compare to **d**). **f** CS setae are arranged in 3 rows on the tarsus, dCS, mCS and vCS (dorsal, medial and ventral, respectively) but widely spaced. There is a ring of CS setae at the tip of the tarsus. Similar to L1, three large MS setae appear in fixed positions (two on the tibia and one on the patella). There are no CS setae on the patella, femur and trochanter. A ventral and a dorsal row of CS sensilla are visible on the coxa in addition to two dense setae fields. Scale bar in **a**: 50 μm in **a**; 120 μm in **b**–**d**; 90 μm in **e**

Similar to L1, the first pedipalp CS setae appear in the 1st instar at the distal tip of the tarsus. Furthermore, the first tibial CS is located on the dorsal-posterior side between the landmark MS setae, which are also present on the pedipalp (Fig. 8a–d). In contrast to L1, the number of pedipalpal CS setae does not significantly increase in the postembryonic stages (Fig. 8c–h) and the arrangement into the four longitudinal rows (dorsal, anterior-medial, posterior-medial and ventral) is only visible in the proximal part of the adult female tarsus (Fig. 9a, f). Although the overall number of tarsal CS setae is similar in males and females (Fig. 2; Suppl. Table 1), the pattern is not comparable due to the bulb-like expansion of tibia and tarsus in the 5th instar and the elaborate shape in the adult (Suppl. Fig. 4c, d). The number of CS setae on the tibia remains constant at one to two throughout postembryonic development and in the adult (Figs. 8b, d, h, 9a, and 2; Suppl. Table 1). We did not detect any CS setae on the patella, femur and trochanter; however, the coxa exhibits a ventral and a dorsal longitudinal row of CS setae along the proximo-distal axis (Fig. 9c, d, f; Suppl. Fig. 3b), both in males and females.

In addition, the anterior pedipalpal coxa has a proximal and a distal field of setae, which we could not classify (purple rings in Fig. 9d–f; Suppl. Fig. 4a). The large distal area consists of a dense field of long, slender, curved setae (Fig. 9d, e; Suppl. Fig. 4a). The setae in the proximal field have a similar structure but are slightly bigger and more spaced. Future electron microscopic analysis of the structure of these setae should reveal if they might function as mechano- or chemoreceptors and if they represent uniform populations.

There is less information available on the distribution of CS setae on spider pedipalps compared to walking legs but a comparison of *P. tepidariorum* to two other spider species, *Argiope bruennichi* (Ganske and Uhl 2018) and *Araneus diadematus* (Foelix 1970), shows again some conserved features. CS setae are mainly located on the tarsus and the coxa. The remaining podomeres show few scattered CS setae only. In all three species, dense setae fields are present on the coxa, which are surrounded by rows of CS setae. The dense setae fields seem to contain CS setae but they have not been analysed in detail, yet (Ganske and Uhl 2018; Foelix 1970).

### The tarsal organ shows a conserved position on the dorsal tarsus

We identified a single tarsal organ on the dorsal-posterior tarsus of the pedipalps and L1 in all postembryonic and adult stages of both sexes (Figs. 1i and 8a–h; Suppl. Fig. 1a-d). This is in line with previous publications and seems to be a conserved feature of the sensory equipment of spiders. Blumenthal (1935), who first described the organ, reported it in 500 spider species. In *P. tepidariorum*, the tarsal organ has the same dome-like shape as has been described for *A. diadematus*, *A. bruennichi* and *C. salei*, for example. Similar to *A. bruennichi* and *A. diadematus*, the tarsal organ of *P. tepidariorum* is located in the proximal half of the tarsus. This is in contrast to *C. salei*, where it is positioned in the distal tarsus next to the claws (Tichy and Barth 1992).

### Trichobothria are located in fixed positions on the tibia and metatarsus

The first couple of trichobothria appear in the 1st instar between the large proximal and distal tibial MS on the posterior-dorsal side (Fig. 4a) and the proximal posterior side of the metatarsus of L1 (Fig. 6a; Suppl. Fig. 1d). In the 2nd instar, a second tibial trichobothrium is visible next to the previous one (Fig. 4b), while the number does not increase on the metatarsus throughout postembryonic stages and in the adult (Fig. 6a–h). In the 3rd instar, an additional trichobothrium is visible on the anterior-dorsal side of the tibia and the number increases to two to three posterior-dorsal and two anterior-dorsal trichobothria in the 4th instar (Fig. 4c–g). Three to four trichobothria are visible on the posterior side of 5th instar males and females (Fig. 4h–k). In subadult females, five tibial trichobothria are located on the posterior-dorsal side and three on the anterior dorsal side of L1 (Fig. 5a, b). The number is about the same in subadult males (four to five trichobothria on the posterior side and three on the anterior side; Suppl. Fig. 3a,b). The final number of tibial trichobothria is 12 in adult females, 6 on the posterior-dorsal side and 6 on the anterior-dorsal side (Fig. 5c–f). In adult males, the number of tibial trichobothria is the same as in females on the posterior side; however, there are only three trichobothria on the anterior dorsal side of the tibia. In the pedipalp, trichobothria can only be seen on the tibia (as the metatarsus is absent). There are only two trichobothria on the adult pedipalps of both males and females. The first one appears in the 1st instar on the posterior side in the middle of the tibial podomere (Fig. 8a). The second trichobothrium appears next to the first one in the 4th instar (Fig. 8d).

Compared to other spider species, from which data are available, the number of trichobothria is lower in *P. tepidariorum*. For example, there are six trichobothria on the male pedipalp in *A. bruennichi* and 12 on the female pedipalpal tibia. However, similar to *A. bruennichi*, there is only a single metatarsal trichobothrium in L1 and about the same number of tibial trichobothria (15 in *A. bruennichi* females, 12 in *P. tepidariorum*). In contrast to other spider species, the trichobothria are not clustered in *P. tepidariorum*, rather, they are aligned in an offset dorsal row (Suppl. Fig. 3e). *C. salei*, for example, exhibits 100 trichobothria per leg, clustered in groups of 3–24. In addition, in some species, trichobothria are located on additional podomeres (patella, tarsus) (Barth 2001; Ganske and Uhl 2018).

### Development and distribution of slit sensilla and lyriform organs

**Fig. 10:**
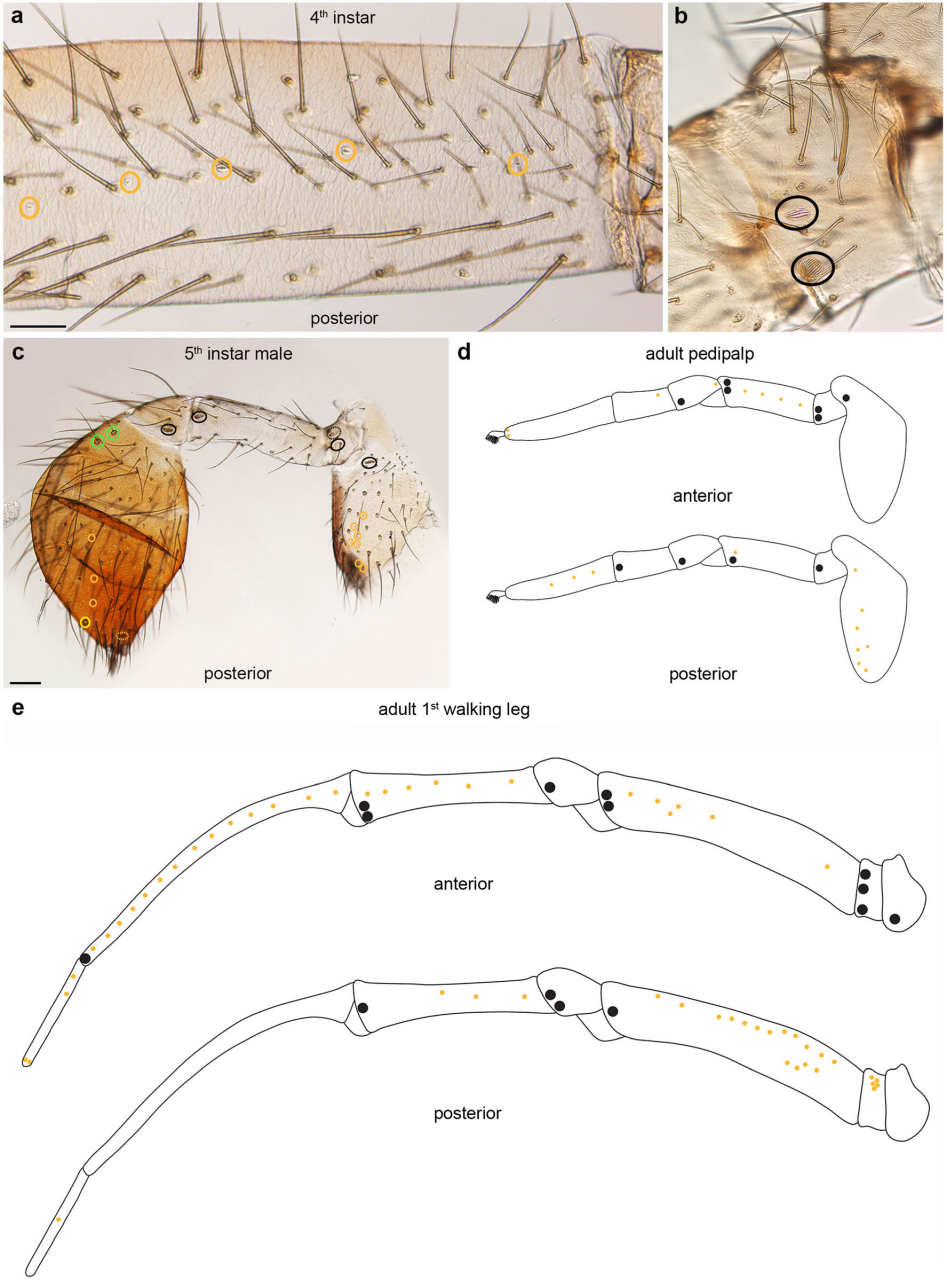
Distribution of lyriform and slit sense organs on the pedipalp and 1st walking leg. Light micrographs (**a**–**c**) and schematic drawings (**d**, **e**); orientations of the preparations/schemes as indicated; dorsal is towards the top; colouring of the rings is the same as in Fig. 3. **a** In the 4th instar, small slit sensilla are arranged in a longitudinal row on the femur. Lyriform organs are located close to the distal joints in the podomeres where they are present. **b** In the pedipalp, slit sense organs are mainly present on the coxa and tarsus. Lyriform organs are located close to the distal joints, similar to L1. **c** The scheme represents female adult pedipalps (anterior and posterior views) and shows the distribution of slit and lyriform organs. The pattern is similar in males and females despite the different morphology. **e** The scheme represents the adult male and female L1. Lyriform organs are present at each joint and slit sense organs are arranged in longitudinal rows except for the trochanter, which has a cluster of 5 slit sense organs. Scale bars: a, 60 micrometers in a, b; c, 60 micrometers: 60 μm

Slit sensilla are present from the 1st larval stage onward, initially only in the distal podomeres (patella, tibia, metatarsus, tarsus). Most slit sensilla cannot be identified individually throughout development due to a lack of landmarks and intercalary growth of the appendages. However, two slit sensilla located at the distal tip of the tarsus are visible in all postembryonic stages and in the adult, both in L1 and the pedipalps (Figs. 10c–e and 2; Suppl. Table 1). Up to the 3rd instar, there are not more than two to four slit sensilla present in each L1 podomere. In the 4th and 5th instars, there is a significant increase of slit sensilla in the femur and metatarsus of L1, which exhibit the highest number of slit sensilla in the adult (22 and 14 on average, respectively; Suppl. Fig. 5b). The slit sensilla are arranged in longitudinal rows along the proximo-distal axis on the anterior and posterior side of the femur and tibia and on the anterior side only on the metatarsus (Fig. 10a, e). In contrast, the trochanter has a single cluster of five slit sensilla (Fig. 10e). We did not detect slit sensilla in the coxa of L1. Similar to L1, there are only few slit sensilla (one to two) on the pedipalps of first instars. However, a significant increase in numbers can only be detected in the coxa towards the end of postembryonic development (Figs. 10e and 2; Suppl. Fig. 5a,b; Suppl. Table 1).

The distribution of the slit sensilla in *P. tepidariorum* is similar to the pattern in *C. salei* and *A. bruennichi* (Barth and Libera 1970; Ganske and Uhl 2018), where these sense organs are also mainly arranged in longitudinal rows along the proximo-distal axis and are located on the lateral sides of the appendages. In addition, all three spider species exhibit a posterior-ventral cluster of slit sense organs on the pedipalpal coxa and a large cluster on the L1 trochanter (Barth and Libera 1970; Ganske and Uhl 2018). Both *P. tepidariorum* and *C. salei* show the additional posterior-ventral row on the femur (Fig. 9e; Barth and Libera 1970). All three species also have considerably fewer slit sense organs on the adult pedipalpal femur compared to L1 (Barth and Libera 1970; Ganske and Uhl 2018).

Lyriform organs show a fixed pattern and are located close to the distal joints of all podomeres where they are present. The pattern is similar in male and female legs and pedipalps despite differences in the length and morphology of the appendages. Almost the complete adult set of lyriform organs is visible in the 1st instar on L1 (one on the coxa, two each on the trochanter, femur, patella and tibia and one on the metatarsus) (Fig. 2; Suppl. Fig. 5; Suppl. Table 1). Additional lyriform organs appear not later than the 5th instar (one each on trochanter, femur, patella and tibia) (Fig. 10b, e; Suppl. Fig. 5b). There are no lyriform organs on the tarsus of L1 (Fig. 10e). In the pedipalp, one lyriform organ each is present on the distal joints of the coxa to patella in the 1st instar (Fig. 2; Suppl. Fig. 5a; Suppl. Table 1). About half of the pedipalps have one lyriform organ at the tibia-tarsus joint (Fig. 2; Suppl. Table 1). Analysis of later stages, in which males and females can be distinguished, reveals that a single lyriform organ is present on the distal tibia in females but not in males Fig. 10c, d; Suppl. Fig. 5a). Similar to L1, the adult set of lyriform organs develops not later than the 5th instar (one on the coxa, three each on trochanter and femur, two on the patella and one on the female tibia; Suppl. Fig. 5a). Similar to L1, there are no lyriform organs on the pedipalpal tarsus.

Compared to *C. salei* and *A. bruennichi* (Barth and Libera 1970; Ganske and Uhl 2018), the number of lyriform organs is surprisingly consistent in the pedipalps (ten in *C. salei*, seven in *A. bruennichi* and nine to ten in *P. tepidariorum*) and in L1 (15 in *C. salei*, and 14 each in *A. bruennichi* and *P. tepidariorum*) of *P. tepidariorum*, considering the different sizes of the appendage joints. In all three spider species, there is a single lyriform organ on the coxa and on the metatarsus of L1 but the numbers vary in the remaining joints. *A. bruennichi* and *P. tepidariorum* also show sex-specific differences in the number of lyriform organs on the tibia of the pedipalps. While there is a lyriform organ at the distal tibial joint in *A. bruennichi* males, the sense organ is missing in females. The reverse is the case in *P. tepidariorum*.

## Conclusion

The external structure of mechano- and chemosensory organs is strongly conserved in *P. tepidariorum* compared to other Entelegynae spider species from which data are available (e.g. Pfreundt and Peters 1981; Foelix 1970; Ganske and Uhl 2018). Similar to other spiders (e.g. Höger and Seyfarth 1995), MS setae show a regular, spaced arrangement in *P. tepidariorum*; however, they do not show a distinctive pattern (e.g. rows) in late postembryonic and adult stages, and cannot be individually identified, except for a few large bristle-like setae. In contrast, all other ESOs seem to appear in fixed positions. CS setae, slit sensilla and trichobothria are arranged in longitudinal rows along the proximal-distal axis. In addition, there is a dense field of CS setae at the tip of the pedipalpal and L1 tarsi and a cluster of slit sense organs on the pedipalpal coxa and L1 trochanter (Barth and Libera 1970; Ganske and Uhl 2018). Lyriform organs are located at the distal joints of all podomeres where they are present and the single tarsal organ is located on the dorsal tarsus. Again, these features are conserved in other Entelegynae spiders (e.g. Barth and Libera 1970; Ganske and Uhl 2018). The invariable pattern suggests that conserved appendage patterning genes define the areas from which the different types of sense organ precursors arise. Furthermore, the spacing might be organized by lateral inhibition mechanisms (Simpson 1990) since the corresponding gene networks have been shown to be involved in sense organ development in spider appendages (Gold et al. 2009; Stollewerk and Seyfarth 2008). We hope that the developmental analysis and established maps will support future comparative analysis of the molecular mechanisms regulating the positioning and identity of the diverse external sense organs of spiders.

## Material and methods

### Spider rearing and handling

*Parasteatoda tepidariorum* spiders were kept and reared as previously described (Mittmann and Wolff 2012; Hilbrant et al. 2012). The spiders for our culture were kindly provided by Alistair McGregor (Oxford Brooks University).

The 1st instar was identified using the staging system by Mittmann and Wolff (2012). To allow a better observation of moults (indicating the onset of the next instar), 1st instar spiders from one cocoon were first separated into groups of 20. After 8 days in smaller groups during which they were fed twice, young spiders were kept in single vials. The emergence of subsequent instars coincided with the shedding of the exuvia which was found at the bottom of the vial. Young spiders were checked almost every day and fed twice a week with *Drosophila melanogaster*.

### Light microscopy imaging and illustration of sense organ maps

For the analysis of the external sense organs, we used either the exuviae, which were preferred because of their transparency, or whole mounts of the spider appendages. Leg and pedipalp preparations of spiders were obtained by placing them for a few minutes at − 20 °C to anaesthetize them. Using forceps and a stereomicroscope, pedipalps and 1st walking legs were removed. The specimens were mounted between two coverslips enabling the imaging from both sides (anterior and posterior). Hoyer’s medium (15 g gum arabic, 25 mL H_2_O, 100 g chloral hydrate, 10 g glycerol) with lactic acid (1:1 lactic acid to Hoyer’s medium) was used as a mounting solution clearing the cuticles of soft tissues and pigments. The mounted samples were placed into an oven at 65 °C overnight. An inverse Leica microscope (DM IL) and corresponding LAS software (version 2.8.1) were used for image acquisition of cuticle preparations.

### SEM sample preparation and imaging

Whole young spiders (1st instar) and the 1st walking legs of adult female spiders (removed from anaesthetized spiders as described above) were fixed in 1:1 heptane and glutaraldehyde/paraformaldehyde (3%) and 0.01% Triton X-100. The detergent reduces the surface tension which enables the samples to submerge. The samples were fixed overnight at 4 °C and dehydrated using an ascending alcohol series (30%, 50%, 70%, 95%, 100%). Samples were incubated for 15′ at each concentration and then chemically dried using HMDS (Sigma-Aldrich) by transferring the samples into a glass dish. The samples were washed once with 1:1100% EtOH and HMDS and replaced with 100% HMDS, which was left in the glass dish for evaporation overnight under the fume hood. Dried samples were carefully mounted onto aluminium stubs prepared with sticky tape. For SEM imaging (FEI Quanta 3DFEG and the FEI Inspect F electron microscopes), the samples were gold sputtered (Agar auto sputter coater model 108A).

### Sense organ quantification and statistical analysis

Light microscope images were used to count the 6 different ESOs on each side of the podomeres (for pedipalp: coxa, trochanter, femur, patella, tibia and tarsus; for L1: coxa, trochanter, femur, patella, tibia, metatarsus, tarsus). For the 1st to 5th instars, we counted the ESOs of 6 appendages each for pedipalps and L1. For the subadult and adult stages, we counted 2 appendages each for pedipalps and L1, and for females and males, respectively.

Microsoft Excel (Office 365, 2019) was used to document data, calculate the mean and standard deviation (SD), as well as for creating bar charts. The schematic drawings are based on light microscopic images and were generated with Inkscape (version 0.92.3) and Adobe Illustrator (version 16.0.0).

## Electronic supplementary material

ESM 1 (PDF 22718 kb)
