# Supplementary material for: Distribution and development of the external sense organ pattern on the appendages of postembryonic and adult stages of the spider Parasteatoda tepidariorum
Source: Dev Genes Evol. 2020 Feb 8;230(2):121–36. doi: 10.1007/s00427-020-00655-8 (PMC7128012; doi:10.1007/s00427-020-00655-8)
Supplement: Supplementary file 1 — (PDF 22718 kb) [file 427_2020_655_MOESM1_ESM.pdf]

Supplementary Material

Supplementary Figure 1

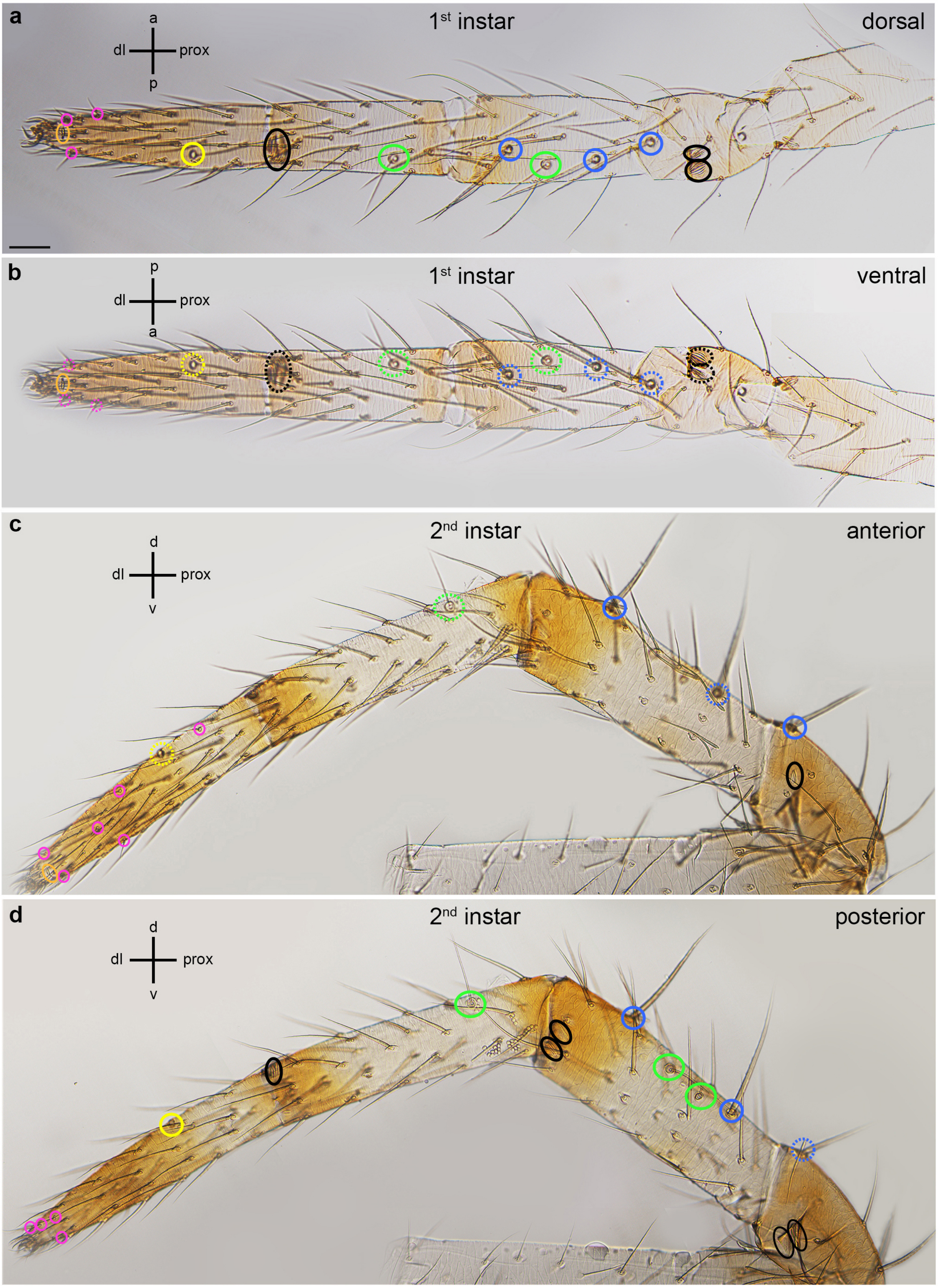

Suppl. Figure 1 Orientations used for describing the distribution of external sense organs. Light micrographs of L1; dorsal, ventral, anterior and posterior views as indicated; dorsal is towards the top. The blue rings indicate the position of large landmark MS setae which are located at unique positions on the patella and tibia throughout postembryonic development and in adults. The green rings indicate trichobothria, the magenta rings CS setae, the orange rings slit sensilla and the black rings lyriform organs. The dashed lines indicate sensilla from the opposite side that are visible due to the transparency of the cuticle. (a-d) All types of sensilla described here are already present in the 1st instar. Preparations shown in the main text are mainly anterior and posterior views. d, dorsal, dl, distal, prox, proximal; v, ventral. Scale bar in a: 30 μm in a-d.

Supplementary Figure 2a

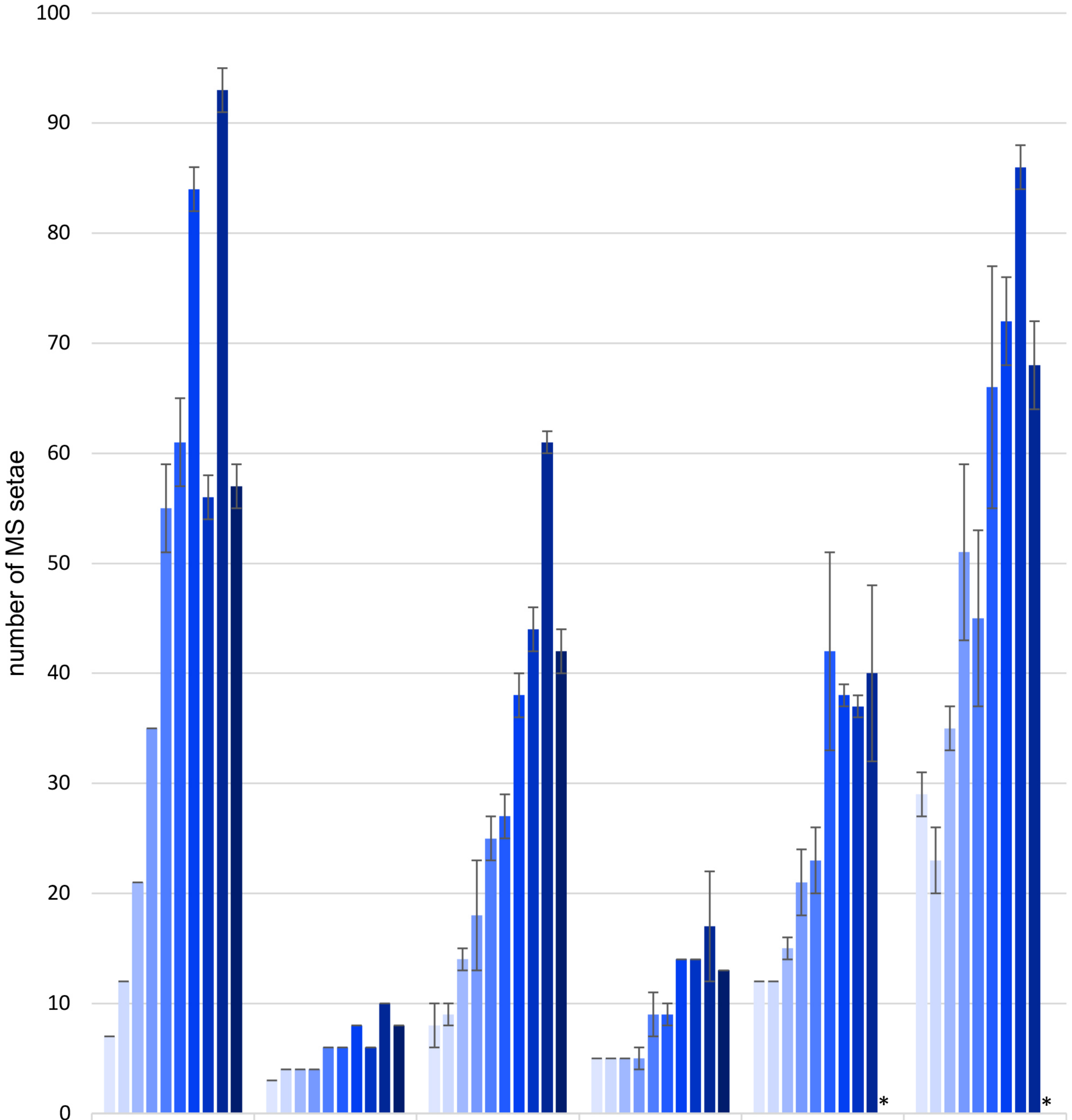

	Coxa	Trochanter	Femur	Patella	Tibia	Tarsus
1	7	3	8	5	12	29
2	12	4	9	5	12	23
3	21	4	14	5	15	35
4	35	4	18	5	21	51
5 ♀	55	6	25	9	23	45
5 ♂	61	6	27	9	42	66
s ♀	84	8	38	14	38	72
s ♂	56	6	44	14	37	86
a ♀	93	10	61	17	40	68
a ♂	57	8	42	13	*	*

Supplementary Figure 2b

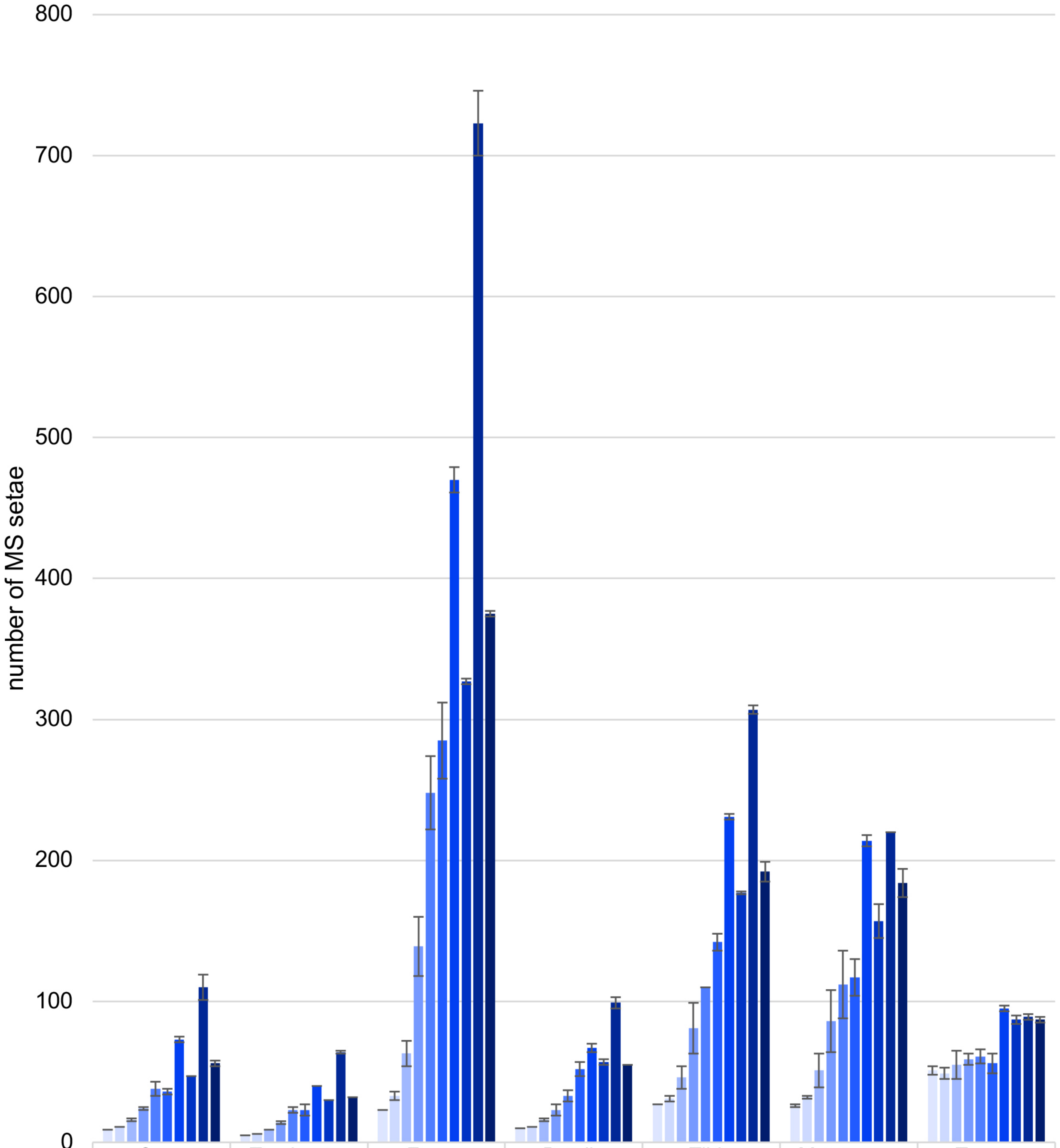

	Coxa	Trochanter	Femur	Patella	Tibia	Metatarsus	Tarsus
1	9	5	23	10	27	26	51
2	11	6	33	11	31	32	49
3	16	9	63	16	46	51	55
4	24	14	139	23	81	86	59
5 ♀	38	23	248	33	110	112	61
5 ♂	36	23	285	52	142	117	56
s ♀	73	40	470	67	231	214	95
s ♂	47	30	327	57	177	157	87
a ♀	110	64	723	99	307	220	89
a ♂	56	32	375	55	192	184	87

Suppl. Figure 2 Distribution of mechanosensory setae. The graph shows the distribution of MS setae on the pedipalp (a) and L1 (b). The number of MS setae continuously increases with each moult. Subadult and adult female spiders have more MS setae than male spiders. On the pedipalp, coxa and tarsus show the largest numbers of MS setae, while the femur exhibits most of the MS setae of the L1 podomeres. We did not count the setae on the tibia and tarsus of the adult male pedipalp (asterisks in a) because these podomeres form a darkly pigmented elaborate copulatory organ which makes the visibility of setae difficult in the light microscope. 1 to 5, 1st to 5th instar; s, subadult; a, adult.

Supplementary Figure 3

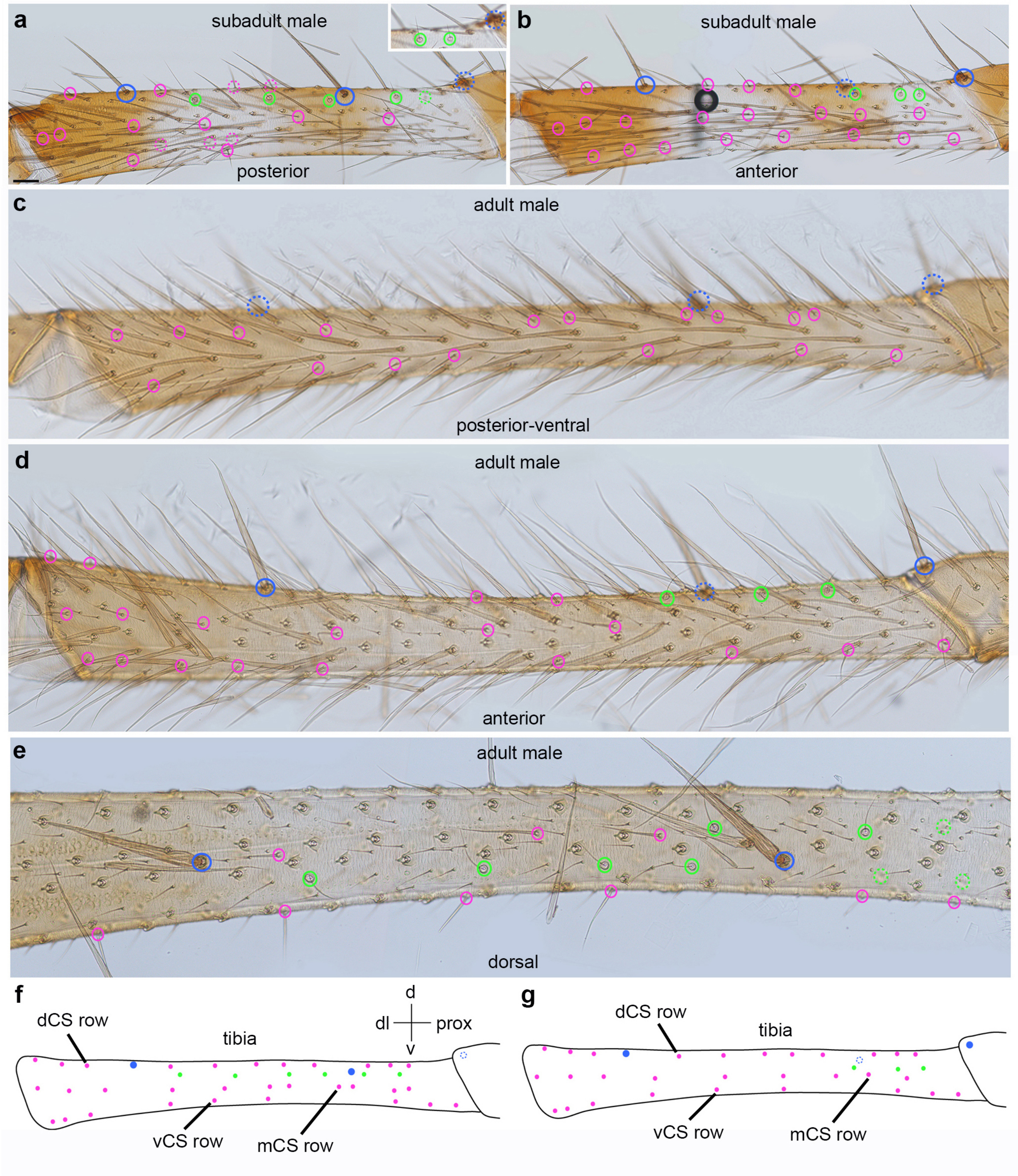

Suppl. Figure 3 Distribution of trichobothria and chemosensory sensilla in the tibia of subadult and adult male 1st walking legs. Light micrographs (a-e) and schematic drawings of the tibia (f,g). Orientation of the preparations, colouring of the rings and abbreviations are the same as in Suppl. Figure 1. (a,b) In the subadult male, the number and distribution of the trichobothria are the same as in the subadult female. Although the CS setae do not occupy the exact same positions, their overall number and arrangement into three rows (dorsal, medial and ventral) is the same as in females. (c) The posterior-ventral view shows that the ventral CS sensilla have intercalated to form a single ventral row in the adult male tibia. (d) In contrast to females, the number of trichobothria (3) remains the same in the

adult male tibia. (e) The dorsal view (anterior towards the top) shows the arrangement of all trichobothria on the tibia - 3 on the dorsal-anterior side and 5 on the posterior-dorsal side. The number and overall arrangement of tibial CS setae are the same in male and female spiders. (f, g) The schemes summarise the distribution of trichobothria and CS setae based on 3 different adult spiders. dCS row, dorsal CS setae row; amCS row, anterior-median CS setae row; pmCS row, posterior-median CS setae row; vCS row, ventral CS setae row. Scale bar in a: 100 μm in a, b; 80 μm in c, d; 70 μm in e.

Supplementary Figure 4

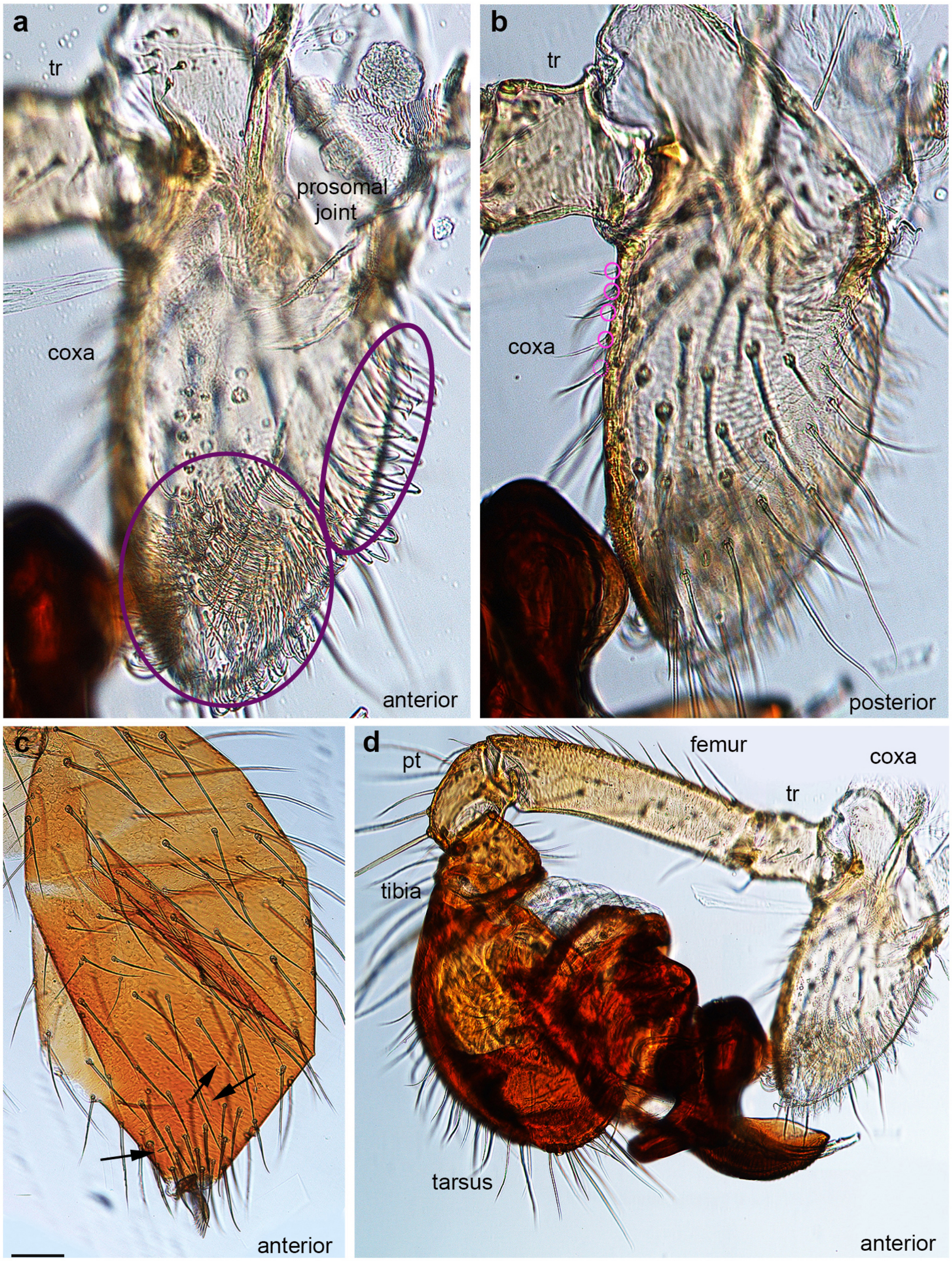

Suppl. Figure 4 Distribution of external sense organs and morphology of the adult male pedipalp. Light micrographs; view of the preparations as indicated, distal is towards the left in a, b and towards the bottom in c, d; *pt*, patella; *tr*, trochanter; colouring of the rings is the same as in Suppl. Figure 1. (a,b) The anterior and posterior view of the coxa show the same pattern and arrangement of the proximal and distal setae fields (purple rings) as well as the dorsal (not in focus) and ventral rows of CS setae (magenta rings) as in the female adult coxa (compare to Figure 8). (c) Tibia and tarsus form a bulb-like shape in the subadult male. The vibrant red colour makes it difficult to discern small slit sense organs (arrows). (d) In the adult male, tibia and tarsus form an elaborate copulatory organ, which is strongly pigmented. Scale bar in c: 50 μm in a, b; 60 μm in c; 100 μm in d.

Supplementary Figure 5a

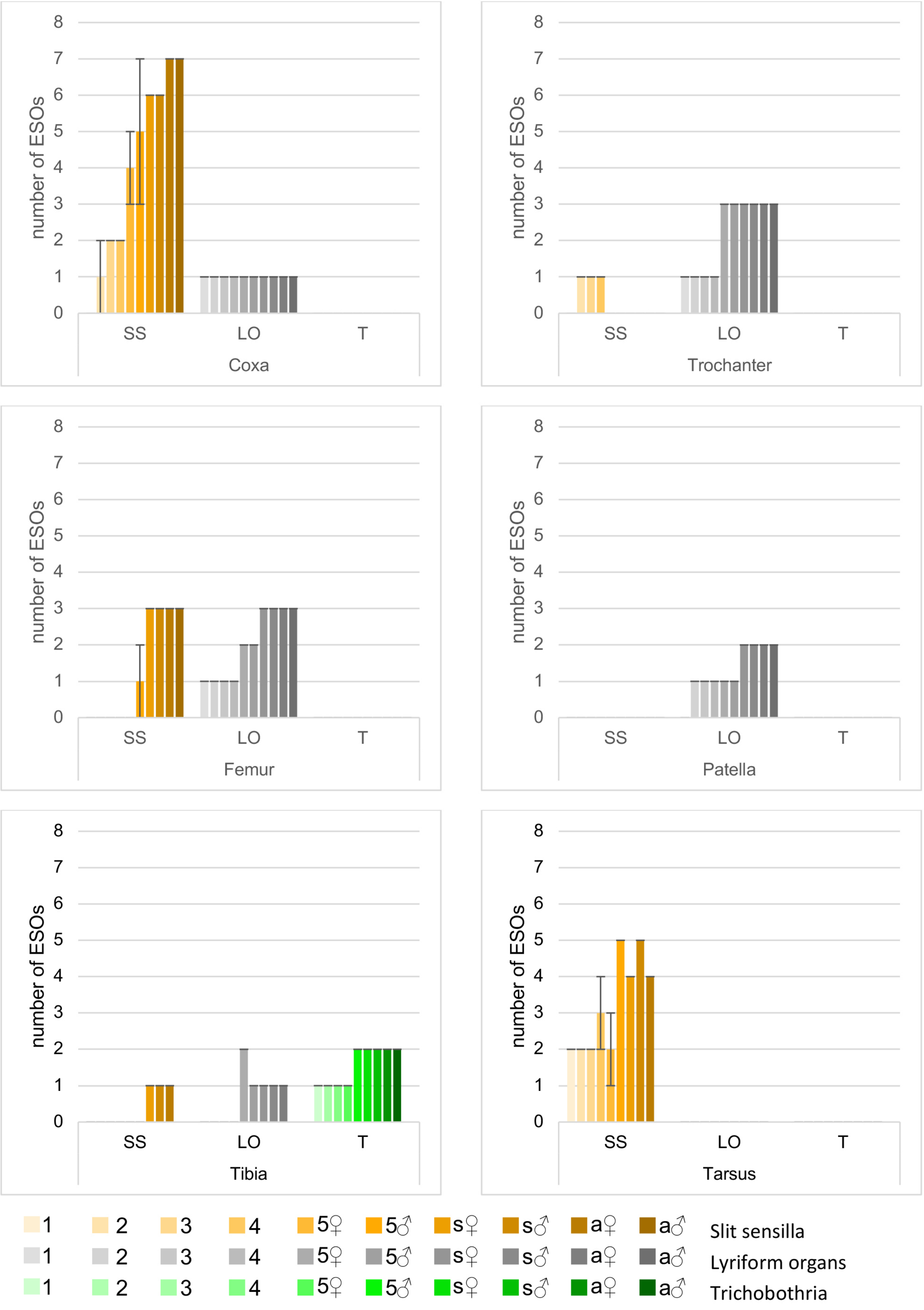

Supplementary Figure 5b

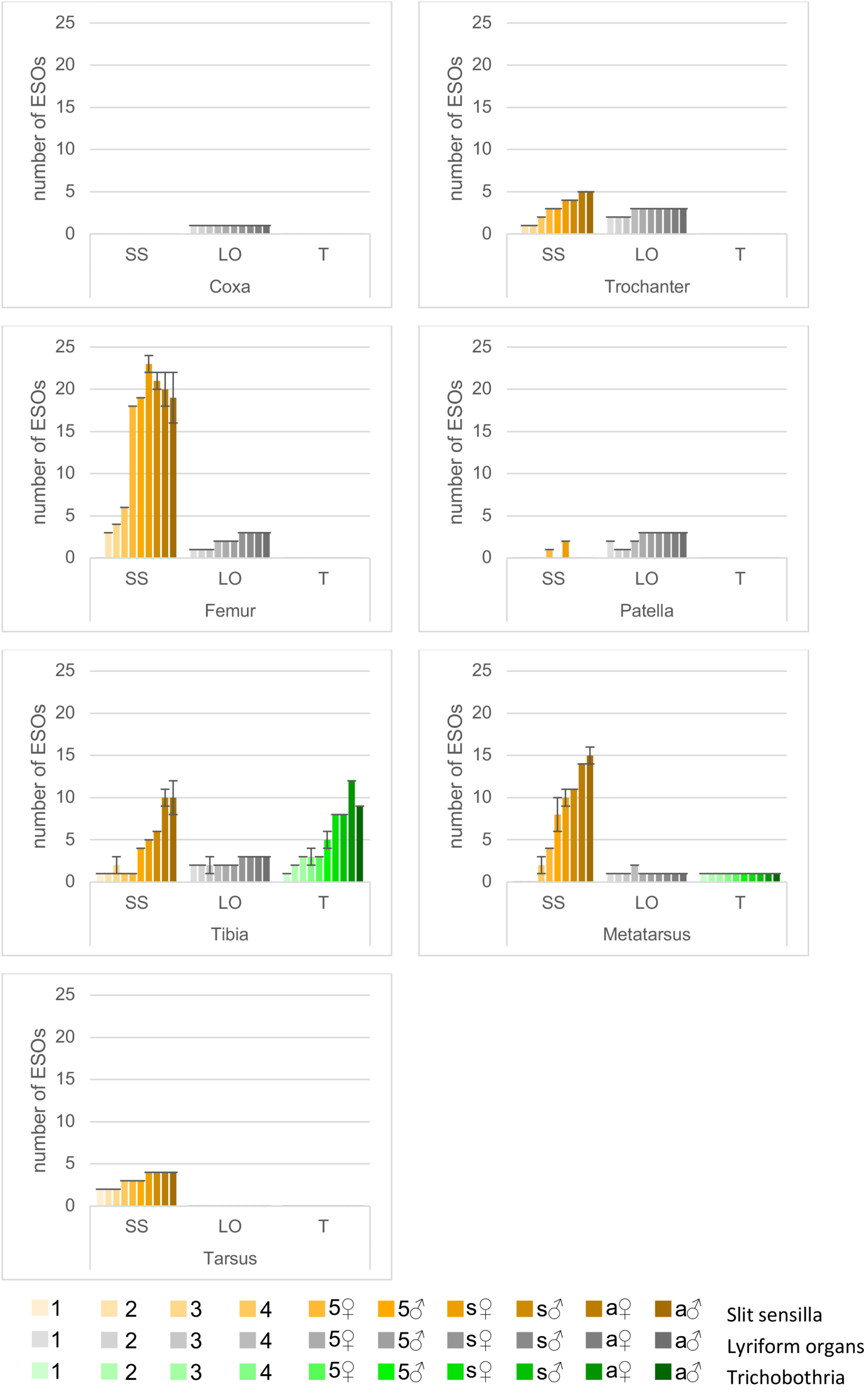

Suppl. Figure 5 Distribution of slit sensilla, lyriform organs and trichobothria. (a) On the pedipalp, coxa and tarsus have the highest numbers of slit sensilla, lyriform organs are not present on the tarsus and trichobothria only occur on the tibia. (b) On L1, the largest number of slit sensilla is present on the femur, followed by the metatarsus. Lyriform organs show a constant number and are visible in all podomers except for the tarsus. While the trichobothria on the metatarsus exhibit a constant number on the metatarsus throughout all stages, the number increases on the tibia and is higher in the female spider. 1 to 5, 1st to 5th instar; s, subadult; a, adult.

Supplemenatary Table 1

Pp	Mechanosensory setae																			
	1st		2nd		3rd		4th		5th ♀		5th ♂		subadult ♀		subadult ♂		adult ♀		adult ♂	
	mean	SD	mean	SD	mean	SD	mean	SD	mean	SD	mean	SD	mean	SD	mean	SD	mean	SD	mean	SD
Coxa	7	0	12	0	21	0	35	0	55	4	61	4	84	2	56	2	93	2	57	2
Trochanter	3	0	4	0	4	0	4	0	6	0	6	0	8	0	6	0	10	0	8	0
Femur	8	2	9	1	14	1	18	5	25	2	27	2	38	2	44	2	61	1	42	2
Patella	5	0	5	0	5	0	5	1	9	2	9	1	14	0	14	0	17	5	13	0
Tibia	12	0	12	0	15	1	21	3	23	3	42	9	38	1	37	1	40	8	0	0
Tarsus	29	2	23	3	35	2	51	8	45	8	66	11	72	4	86	2	68	4	0	0

Pp	Chemosensory setae																			
	1st		2nd		3rd		4th		5th ♀		5th ♂		subadult ♀		subadult ♂		adult ♀		adult ♂	
	mean	SD	mean	SD	mean	SD	mean	SD	mean	SD	mean	SD	mean	SD	mean	SD	mean	SD	mean	SD
Coxa	1	0	2	0	3	0	5	0	5	1	7	1	15	0	16	0	16	0	17	0
Trochanter	0	0	0	0	0	0	0	0	0	0	0	0	0	0	0	0	0	0	0	0
Femur	0	0	0	0	0	0	0	0	0	0	0	0	0	0	0	0	0	0	0	0
Patella	0	0	0	0	0	0	0	0	0	0	0	0	0	0	0	0	0	0	0	0
Tibia	0	0	0	0	0	0	1	0	1	1	1	0	1	0	1	0	1	0	0	0
Tarsus	2	0	8	1	11	5	10	1	12	2	11	2	23	1	15	1	28	4	0	0

Pp	Slit sensilla																			
	1st		2nd		3rd		4th		5th ♀		5th ♂		subadult ♀		subadult ♂		adult ♀		adult ♂	
	mean	SD	mean	SD	mean	SD	mean	SD	mean	SD	mean	SD	mean	SD	mean	SD	mean	SD	mean	SD
Coxa	0	0	1	1	2	0	2	0	4	1	5	2	6	0	6	0	7	0	7	0
Trochanter	0	0	1	0	1	0	1	0	0	0	0	0	0	0	0	0	0	0	0	0
Femur	0	0	0	0	0	0	0	0	0	0	1	1	3	0	3	0	3	0	3	0
Patella	0	0	0	0	0	0	0	0	0	0	0	0	0	0	0	0	0	0	0	0
Tibia	0	0	0	0	0	0	0	0	0	0	0	0	1	0	1	0	1	0	0	0
Tarsus	2	0	2	0	2	0	3	1	2	0	5	1	4	0	5	0	4	0	0	0

Pp	Lyriform organs																			
	1st		2nd		3rd		4th		5th ♀		5th ♂		subadult ♀		subadult ♂		adult ♀		adult ♂	
	mean	SD	mean	SD	mean	SD	mean	SD	mean	SD	mean	SD	mean	SD	mean	SD	mean	SD	mean	SD
Coxa	1	0	1	0	1	0	1	0	1	0	1	0	1	0	1	0	1	0	1	0
Trochanter	1	0	1	0	1	0	1	0	3	0	3	0	3	0	3	0	3	0	3	0
Femur	1	0	1	0	1	0	1	0	2	0	2	0	3	0	3	0	3	0	3	0
Patella	0	0	1	0	1	0	1	0	1	0	1	0	2	0	2	0	2	0	2	0
Tibia	0	0	0	0	0	0	0	0	2	0	1	0	1	0	1	0	1	0	0	0
Tarsus	0	0	0	0	0	0	0	0	0	0	0	0	0	0	0	0	0	0	0	0

Pp	Trichobothria																			
	1st		2nd		3rd		4th		5th ♀		5th ♂		subadult ♀		subadult ♂		adult ♀		adult ♂	
	mean	SD	mean	SD	mean	SD	mean	SD	mean	SD	mean	SD	mean	SD	mean	SD	mean	SD	mean	SD
Coxa	0	0	0	0	0	0	0	0	0	0	0	0	0	0	0	0	0	0	0	0
Trochanter	0	0	0	0	0	0	0	0	0	0	0	0	0	0	0	0	0	0	0	0
Femur	0	0	0	0	0	0	0	0	0	0	0	0	0	0	0	0	0	0	0	0
Patella	0	0	0	0	0	0	0	0	0	0	0	0	0	0	0	0	0	0	0	0
Tibia	1	0	1	0	1	0	1	0	2	0	2	0	2	0	2	0	2	0	0	0
Tarsus	0	0	0	0	0	0	0	0	0	0	0	0	0	0	0	0	0	0	0	0

Supplemenatary Table 1 - continued

Mechanosensory setae

L1	1st		2nd		3rd		4th		5th ♀		5th ♂		subadult ♀		subadult ♂		adult ♀		adult ♂	
	mean	SD	mean	SD	mean	SD	mean	SD	mean	SD	mean	SD	mean	SD	mean	SD	mean	SD	mean	SD
Coxa	9	0	11	0	16	1	24	1	38	5	36	2	73	2	47	0	110	9	56	2
Trochanter	5	0	6	0	9	0	14	1	23	2	23	4	40	0	30	0	64	1	32	0
Femur	23	0	33	3	63	9	139	21	248	26	285	27	470	9	327	2	723	23	375	2
Patella	10	0	11	0	16	1	23	4	33	4	52	5	67	3	57	2	99	4	55	0
Tibia	27	0	31	2	46	8	81	18	110	0	142	6	231	2	177	1	307	3	192	7
Metatarsus	26	1	32	1	51	12	86	22	112	24	117	13	214	4	157	12	220	0	184	10
Tarsus	51	3	49	4	55	10	59	4	61	5	56	7	95	2	87	3	89	2	87	2

Chemosensory setae

L1	1st		2nd		3rd		4th		5th ♀		5th ♂		subadult ♀		subadult ♂		adult ♀		adult ♂	
	mean	SD	mean	SD	mean	SD	mean	SD	mean	SD	mean	SD	mean	SD	mean	SD	mean	SD	mean	SD
Coxa	0	0	0	0	0	0	0	0	0	0	0	0	0	0	0	0	0	0	0	0
Trochanter	0	0	0	0	0	0	0	0	0	0	0	0	0	0	0	0	0	0	0	0
Femur	0	0	0	0	0	0	0	0	0	0	0	0	0	0	0	0	0	0	0	0
Patella	0	0	0	0	0	0	1	1	1	0	3	1	3	0	3	0	5	0	4	0
Tibia	0	0	1	0	2	0	7	4	14	5	16	7	47	8	28	2	45	4	43	4
Metatarsus	0	0	0	0	3	0	13	4	37	4	43	7	49	2	50	2	73	4	67	3
Tarsus	7	1	12	2	17	5	30	1	36	7	41	6	57	2	57	3	60	3	61	0

Slit sensilla

L1	1st		2nd		3rd		4th		5th ♀		5th ♂		subadult ♀		subadult ♂		adult ♀		adult ♂	
	mean	SD	mean	SD	mean	SD	mean	SD	mean	SD	mean	SD	mean	SD	mean	SD	mean	SD	mean	SD
Coxa	0	0	0	0	0	0	0	0	0	0	0	0	0	0	0	0	0	0	0	0
Trochanter	0	0	1	0	1	0	2	0	3	0	3	0	4	0	4	0	5	0	5	0
Femur	0	0	3	0	4	0	6	2	18	2	19	0	23	1	21	1	20	2	19	3
Patella	0	0	0	0	0	0	0	0	1	0	0	0	2	0	0	0	0	0	0	0
Tibia	1	0	1	0	2	1	1	0	1	0	4	0	5	0	6	0	10	1	10	2
Metatarsus	0	0	0	0	0	0	2	1	4	0	8	2	10	1	11	0	14	0	15	1
Tarsus	2	0	2	0	2	0	3	0	3	0	3	0	4	0	4	0	4	0	4	0

Lyriform organs

L1	1st		2nd		3rd		4th		5th ♀		5th ♂		subadult ♀		subadult ♂		adult ♀		adult ♂	
	mean	SD	mean	SD	mean	SD	mean	SD	mean	SD	mean	SD	mean	SD	mean	SD	mean	SD	mean	SD
Coxa	1	0	1	0	1	0	1	0	1	0	1	0	1	0	1	0	1	0	1	0
Trochanter	2	0	2	0	2	0	3	0	3	0	3	0	3	0	3	0	3	0	3	0
Femur	1	0	1	0	1	0	2	0	2	0	2	0	3	0	3	0	3	0	3	0
Patella	2	0	1	0	1	0	2	0	3	0	3	0	3	0	3	0	3	0	3	0
Tibia	2	0	2	0	2	1	2	0	2	0	2	0	3	0	3	0	3	0	3	0
Metatarsus	1	0	1	0	1	0	2	0	1	0	1	0	1	0	1	0	1	0	1	0
Tarsus	0	0	0	0	0	0	0	0	0	0	0	0	0	0	0	0	0	0	0	0

Trichobothria

L1	1st		2nd		3rd		4th		5th ♀		5th ♂		subadult ♀		subadult ♂		adult ♀		adult ♂	
	mean	SD	mean	SD	mean	SD	mean	SD	mean	SD	mean	SD	mean	SD	mean	SD	mean	SD	mean	SD
Coxa	0	0	0	0	0	0	0	0	0	0	0	0	0	0	0	0	0	0	0	0
Trochanter	0	0	0	0	0	0	0	0	0	0	0	0	0	0	0	0	0	0	0	0
Femur	0	0	0	0	0	0	0	0	0	0	0	0	0	0	0	0	0	0	0	0
Patella	0	0	0	0	0	0	0	0	0	0	0	0	0	0	0	0	0	0	0	0
Tibia	1	0	2	0	3	0	3	1	3	0	5	1	8	0	8	0	12	0	9	0
Metatarsus	1	0	1	0	1	0	1	0	1	0	1	0	1	0	1	0	1	0	1	0
Tarsus	0	0	0	0	0	0	0	0	0	0	0	0	0	0	0	0	0	0	0	0

Suppl. Table 1 Number of external sensory sensilla on the pedipalps and 1st walking legs. The table shows the average number of MS and CS setae, slit sensilla, lyriform organs and trichobothria in all postembryonic and adult stages for both male and female spiders. Tarsal organs are not included in the table since there is only one each on the pedipalpal and L1 tarsi.
